# Estimates of the global burden of Congenital Rubella Syndrome, 1996-2019

**DOI:** 10.1016/j.ijid.2023.09.003

**Published:** 2023-12

**Authors:** Emilia Vynnycky, Jennifer K. Knapp, Timos Papadopoulos, Felicity T. Cutts, Masahiko Hachiya, Shinsuke Miyano, Susan E. Reef

**Affiliations:** 1 Statistics Modelling and Economics Department, United Kingdom Health Security Agency, London, UK; 2 Department of Infectious Disease Epidemiology, London School of Hygiene & Tropical Medicine, London, UK; 3 TB Modelling Group and Centre for Mathematical Modelling of Infectious Diseases, London School of Hygiene & Tropical Medicine, London, UK; 4 Global Immunization Division, Centers for Disease Control and Prevention, Atlanta, Georgia, USA; 5 Bureau of International Health Cooperation, National Center for Global Health and Medicine, Toyama, Shinjuku-ku, Tokyo, Japan

**Keywords:** Rubella, Measles-rubella vaccine, Congenital Rubella Syndrome, Mathematical modeling, Seroprevalence

## Abstract

**Objectives:**

Many countries introduced rubella-containing vaccination (RCV) after 2011, following changes in recommended World Health Organization (WHO) vaccination strategies and external support. We evaluated the impact of these introductions.

**Methods:**

We estimated the country-specific, region-specific, and global Congenital Rubella Syndrome (CRS) incidence during 1996-2019 using mathematical modeling, including routine and campaign vaccination coverage and seroprevalence data.

**Results:**

In 2019, WHO African and Eastern Mediterranean regions had the highest estimated CRS incidence (64 [95% confidence intervals (CI): 24-123] and 27 [95% CI: 4-67] per 100,000 live births respectively), where nearly half of births occur in countries that have introduced RCV. Other regions, where >95% of births occurred in countries that had introduced RCV, had a low estimated CRS incidence (<1 [95% CI: <1 to 8] and <1 [95% CI: <1 to 12] per 100,000 live births in South-East Asia [SEAR] and the Western Pacific [WPR] respectively, and similarly in Europe and the Americas). The estimated number of CRS births globally declined by approximately two-thirds during 2010-2019, from 100,000 (95% CI: 54,000-166,000) to 32,000 (95% CI: 13,000-60,000), representing a 73% reduction since 1996, largely following RCV introductions in WPR and SEAR, where the greatest reductions occurred.

**Conclusions:**

Further reductions can occur by introducing RCV in remaining countries and maintaining high RCV coverage.

## Highlights

• Many countries introduced rubella-containing vaccination (RCV) after 2010.
• The global Congenital Rubella Syndrome (CRS) incidence fell by 66% during 2010-2019.
• By 2019, 32,000 (95% confidence intervals: 13,000-60,000) babies were born annually with CRS globally.
• The biggest reductions were in South-East Asia and the Western Pacific region.
• Further declines in CRS incidence need continued high coverage and RCV in all nations.

## Introduction

Congenital Rubella Syndrome (CRS) is associated with significant disability, including congenital heart defects, cataracts, hearing impairment, and developmental delay [1]. When a woman is infected with rubella in early pregnancy, the infant may be born with CRS. The CRS burden is typically low in countries where coverage with rubella-containing vaccines (RCVs) is high. Previous estimates [2] suggest that during 1996-2010, the number of children born globally with CRS decreased modestly from 119,000 to just over 100,000. Much of the estimated CRS burden in 2010 was in Africa and South-East Asia where rubella vaccination had not yet been introduced in 96% and 64% of countries [2]. Since 2010, the World Health Organization (WHO) recommended all countries introduce the rubella vaccine and suggested a preferred strategy of a campaign targeting a wide age range [3], followed immediately by inclusion in the childhood immunization schedule. The Global Alliance for Vaccines and Immunization (GAVI) has also begun funding the introduction of RCV in many low-income countries [4]. In total, Over forty countries have since introduced RCV, and new estimates of the burden of CRS are needed to quantify the impact of their introduction [2].

Updated estimates of the pre-COVID era CRS burden are also needed for monitoring the impact of pandemic-related disruption. Recent estimates suggest that the COVID pandemic has disrupted immunization delivery services, with approximately 27.2 million (95% CI: 23.4-32.5) children missing their first dose of measles-containing vaccine (MCV) in 2019 [5] and an estimated 7.9% reduction in the global MCV coverage in 2020 compared to that expected. Since RCV is administered together with MCV, prolonged disruption of immunization services could lead to increasing proportions of girls still being susceptible to infection when reaching child-bearing age, and hence an increased CRS burden.

In this paper, we update a previously published literature review of rubella seroprevalence studies [2] and estimate the global CRS burden during 1996-2019 using mathematical modeling.

## Methods

### Data sources

#### Literature search to identify seroprevalence data

Our previous systematic review [2] covered 1990-December 2011. In our updated review, we identified age-stratified rubella seroprevalence data published between January 2012 and September 2020. We scanned citations in published papers and published [6] and unpublished literature reviews (Winter et al, personal communication) and a co-author's (JK Knapp) archive for additional datasets. We also used an unpublished dataset provided by Centers for Disease Control and Prevention (CDC) from a representative population in Indonesia (S Reef, personal communication).

We followed previously published methods [2,7] to identify datasets from the literature search, reviewing the abstracts to identify potentially relevant articles, before reading the publication in full and extracting age-specific numbers of seropositive and seronegative individuals from eligible datasets, by sex where possible. “Child-bearing” age was assumed to be 15-44 years and equivocal rubella antibody results were interpreted as seropositive. Datasets were eligible for inclusion if they were considered unbiased [2] and collected before RCV had been introduced. For countries without data predating the introduction of RCV, subsequent data collected in age groups that had remained unvaccinated were also eligible. Details of the search and eligibility criteria are provided elsewhere [2].

#### Demographic data

The total population size, age-specific number of females for 1996-2019 by single year and age, and age-specific fertility rates were extracted for each country from United Nations (UN) population databases [8]. Fertility rates were available for 5-year age groups and time periods (1995-2000, 2000-2005, 2005-2010, 2010-2015 and 2015-2020). Annual values for each 5-year age group were interpolated from the corresponding period. For each country, the total number of live births for 1996-2019, the crude birth rate, and age and sex-specific survival data for the period 2015-2020 were also extracted from UN population databases [8].

#### Vaccination data

We used UN population estimates of the annual number of live births in each country and WHO region to calculate the annual percentage of all live births in each WHO region from 1996-2019 which occurred in countries that had introduced RCV.

Countries have submitted annual vaccination coverage data to WHO since 1980 [9]. Since 2000, WHO and the United Nations Children's Fund (UNICEF) jointly reviewed these and available special survey data to obtain the WHO-UNICEF coverage estimates (WUENIC) [9]. For 53 countries reporting having RCV in the national immunization schedule, but lacking RCV coverage data for the relevant years, we assumed that RCV coverage equaled the WUENIC estimate [10] for the first and second doses of MCV (MCV1 and MCV2) or the MCV coverage reported to WHO if the WUENIC estimate was unavailable. Alternative coverage sources were identified if neither was available. For the remaining gaps, the coverage data were interpolated (Supplement Table 1).

Historical data on the target population and the estimated coverage for periodic mass RCV supplementary immunization activities (“SIAs”) available from WHO [11] and elsewhere (Supplement, Section A) were also used. For countries known to vaccinate adolescent girls for given years, we used published coverage data where possible (Supplement, Section A); otherwise, we assumed 50% coverage. In previous analyses [2], assuming either 10% or 90% coverage for adolescent girls did not substantially affect global burden estimates from 1996. Reliable estimates of post-partum or private (but not public) sector vaccination were unavailable. Missing SIAs were supplemented from publications (Supplement, Section A). One country (Uganda) reportedly introduced RCV via an SIA in October 2019. Since this would not have greatly affected the burden in 2019, for simplicity, for Uganda, we assumed zero RCV coverage for 2019.

### Mathematical modeling

#### Overview

We first used catalytic models [12] to analyze the seroprevalence data identified in the literature review. The resulting pre-vaccination force of infection (rate at which susceptible individuals are infected) estimates were used to calculate age-dependent contact parameters, which were then included in an age-structured dynamic transmission model.

#### Catalytic modeling

The seroprevalence datasets identified in the updated literature review were analyzed using four catalytic models (labeled A, B, C, D), as described previously [2,13] to calculate the average age-specific force of infection before RCV had been introduced (Supplement, section B). The catalytic models assumed that the pre-vaccination force of infection differed between those aged ≤13 and >13 years and estimates from the most plausible model were used in CRS burden calculations [2,13]. For one setting (Laos) lacking data predating the introduction of RCV, we used data collected several years after an SIA [14] and restricted the catalytic modeling to data from unvaccinated age groups, and assumed a 50% reduction in the force of infection after the SIA. Alternative assumptions for this reduction did not affect the pre-vaccination force of infection estimated among adults [14].

As in previous analyses, confidence intervals (CIs) on the force of infection and (where applicable) the sensitivity of the assay for each dataset and catalytic model were generated using 1000 bootstrap-derived-seroprevalence datasets [2] using nonparametric bootstrap for binary data, based on 1000 bootstrap datasets, following Shkedy et al [15].

#### The transmission model

The transmission model is similar to the one used previously [2,13]. The population is stratified into those with maternal immunity (lasting 6 months), susceptible, pre-infectious, infectious, and immune through either vaccination or natural infection. The population was further stratified by sex into single-year age groups, with each cohort moving into the subsequent age group at the end of each year, following Schenzle's approach [16]. In contrast with the model used previously, the population was aged 0-99 years and the same general model was used for all countries, irrespective of whether they had introduced vaccination. We used country-specific birth rates and age- and sex-specific death rates fixed at 2015-2020 levels, with the latter calculated using UN population survival data [8]. The model's differential equations, model diagram, and further details are in the Supplement, section C, and in reference [2].

Following previous methods, the force of infection in the transmission model changes over time and is calculated using the number of infectious individuals and the effective contact rate. Contact is described using the following matrix of “Who Acquires Infection From Whom”, whose general structure is based on contact survey data [17]:

$$\left(\begin{array}{cc} \beta_{1} & 0.7\beta_{2}\\ 0.7\beta_{2} & \beta_{2} \end{array}\right)$$

The effective contact rate differs between <13 and ≥13 year olds. For a given country, $\beta_{1}$ and $\beta_{2}$ are calculated from the pre-vaccination force of infection in <13 and ≥13-year-olds, as estimated by fitting catalytic models to country-specific age-stratified rubella seroprevalence data [2] (see above).

For each country, the transmission model was run using 1000 values for the pre-vaccination force of infection, vaccine efficacy, vaccine coverage, and risk that a child born to a mother who was infected with rubella while pregnant had CRS, which were varied in the same range as that used previously [13] (Supplement, Section D and Table 4). The method used to compile the 1000 values for the pre-vaccination force of infection for a given country depended on the number of seroprevalence datasets available for that country (Supplement, Table 9).

The transmission model was used to calculate the CRS incidence per 100,000 live births and the number of CRS cases born annually from 1996-2019, for each country, WHO region, and globally (Supplement, Section E). We also computed the number of CRS cases in the African (AFR), East Mediterranean (EMR), South-East Asian (SEAR), and Western Pacific (WPR) regions and globally if RCV had not been introduced in these areas since 2010. We subtracted the estimated number of CRS cases obtained using the implemented RCV coverage from these numbers to obtain the number of CRS cases that were averted by introducing RCV after 2010. The 95% range of all model outputs, approximating the 95% confidence intervals, was calculated as the 95% range of the 1000 model runs. As previously, given China's large population size, the regional incidence for WPR was calculated with and without excluding China.

## Results

### Literature search and analyses of seroprevalence data

**Figure 1:**
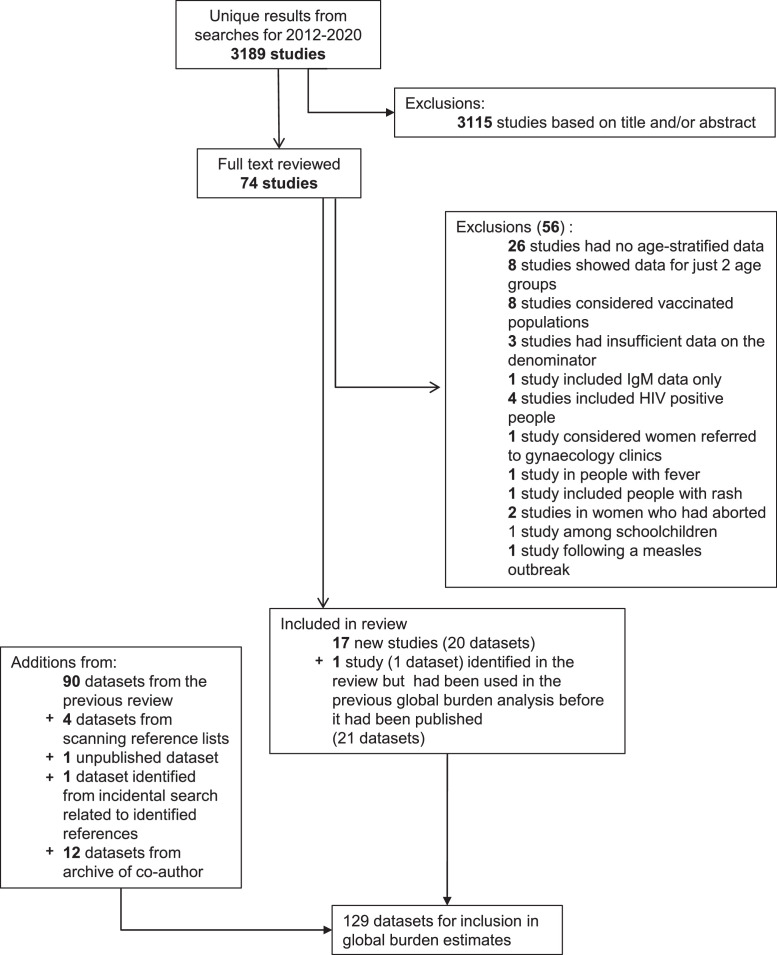
Results of the literature search for age-specific serological data for estimating the force of infection before the introduction of rubella vaccination. The small discrepancy between the number of datasets reported here for the previous review is due to the latter counting several datasets stratified by urban and rural locations as one dataset. In this figure, we refer to a publication as a study.

Figure 1 summarizes the literature search results. After de-duplication and excluding ineligible studies, we identified 21 potential serological datasets for estimating the average pre-vaccination force of infection. Five additional datasets were identified from scanning reference lists or conducting searches related to the identified studies, besides 12 datasets predating 1990 from a co-author's (JKK) archive. Conferring with an unpublished literature review (Winter et al, personal communication) led to no further datasets being identified. After fitting catalytic models, the selected model for one dataset (Nigeria [Keffi [18]] had implausibly low force of infection estimates (Supplement, Table 12) and was dropped from our analyses.

After including the datasets from the previous review [2] and one unpublished study from Indonesia provided by CDC (Susan Reef, personal communication), 129 datasets from 70 countries were available for estimating the global CRS burden (Table 1).

**Table 1:** Summary of the serological datasets collected which were used to estimate the global burden of CRS^a^. Studies not used in the previous update are in bold font. The references can be found in the Supplement.

	Africa	Americas	E Mediterranean	Europe	SE Asia	W Pacific
**No. countries represented**	19	12	12	12	5	10
**No. of countries in region**	47	39	22	51	11	27
**Datasets**	**Algeria, 2005-7;** Benin, 1993; **Burkina Faso, 2007-8; Cameroon (Bafoussam), 2016, Cameroon (Yaounde), <2018;** Congo, <1991; Cote d'Ivoire, 1975 & 1985-6; **Democratic Republic of the Congo (Kikwit, Mikalayi, Tshikapa, Vanga), 2008-9;** Ethiopia, 1981 & 1994; **Ethiopia (Amhara), 2015-17; Ethiopia (Hawassa), 2016;** Gabon, 1985; Ghana, 1997; Kenya, 1996-9 (Kilifi); **Kenya (Eldoret), 2005;** Madagascar, 1990-1995; Mozambique, 2002; **Namibia, 2010;** Nigeria, <1978, <2002 & 2007-8; **Nigeria (Kaduna), 2011-12; Nigeria (Ilorin), 2012, Nigeria (Maiduguri), 2013; Nigeria (Kaduna), 2015;** Senegal, 1996-2001; South Africa, 2003, **South Africa (Soweto), 2014-16; Tanzania (Mwanza), 2012-13;** Zambia, 1979-80	Argentina, 1967-8 (urban & rural) & 1981 (Mar de Plata); Brazil, 1967-8, 1987 & 1996-8; Canada, <1967; Chile 1967-8 (Santiago & rural); **Chile (Santiago), 1983;** Haiti, 2003; Jamaica, 1967-8 (Kingston & rural); Mexico, 1987-88 & 1989; Panama 1967-8 (Panama City & rural); Peru, 1967-8 (Lima & rural) & 2003; Trinidad 1966-7, 1967-8 (Port au Spain & rural); Uruguay, 1967-7 (urban and rural); USA <1967 (Atlanta & Houston)	Bahrain, 1981; **Egypt (Cairo), 1973**; Iran, 1993-95; Jordan, 1982-3; Kuwait, <1978; Lebanon, 1980-1; Morocco, 1969-70; Pakistan, <1997, 1999-2004; Saudi Arabia, 1989 & 1992-93; **Sudan (Khartoum), 2015-16; Sudan (Khartoum), 2016;** Tunisia, <1970; Yemen, 1985 & 2002-03	Czech Republic, <1967 & **Czech Republic, 1984 (Prague)**; Denmark, <1967 &1983; East Germany, 1990; England, <1967 & 1986-7; Finland, 1979; France, <1967; **Kyrgyzstan, 1968-70** and 2001; **Poland, 1969, 1973, 1979 (urban), 1979 (rural), 1982 (urban), 1982 (rural)**; Romania, <1989; **Spain, 1969-71; Switzerland, 1985**; Turkey, 1998, 2003-04 & 2005	Bangladesh, 2004-5; India, 1968 (urban & rural Delhi), 1972-3 (Chandrigarh & Lucknow), 1976 (Calcutta), <1987 (Delhi), <1990 (Delhi), 1999-2000 (urban and rural Vellore); **India (Kerala), 2016; India, 2017; Indonesia, 2007 (*S Reef, personal communication***); Nepal, 2008, Thailand, 1978	Australia (<1967?); **Cambodia, 2012;** China (1979-80); Fiji, <1973; Japan, <1967 (Sapporo & Ohtsu); **Laos, 2014;** Malaysia, <1972; Singapore, 1975-79, Taiwan, 1984 & 1984-6; Central Vietnam, 2009-2010
# datasets (# with SS >1000)	35 (8)	27 (4)	16 (7)	24 (8)	15 (5)	12 (7)

a The year in which the study was carried out is not known for several studies. For these studies, the table includes “<” followed by the year of publication. # = number; CRS = Congenital Rubella Syndrome; SS = sample size.

The percentage of countries with data varied between regions, coming from 55% or fewer of the constituent countries in a region. The dataset size and quality also varied. WPR had the largest percentage of datasets which had a sample size exceeding 1000 individuals (58%, 7 out of 12 datasets), as compared with 23% (8/35) for AFR and 15% (4/27) for the Americas (AMR).

### CRS incidence

**Figure 2:**
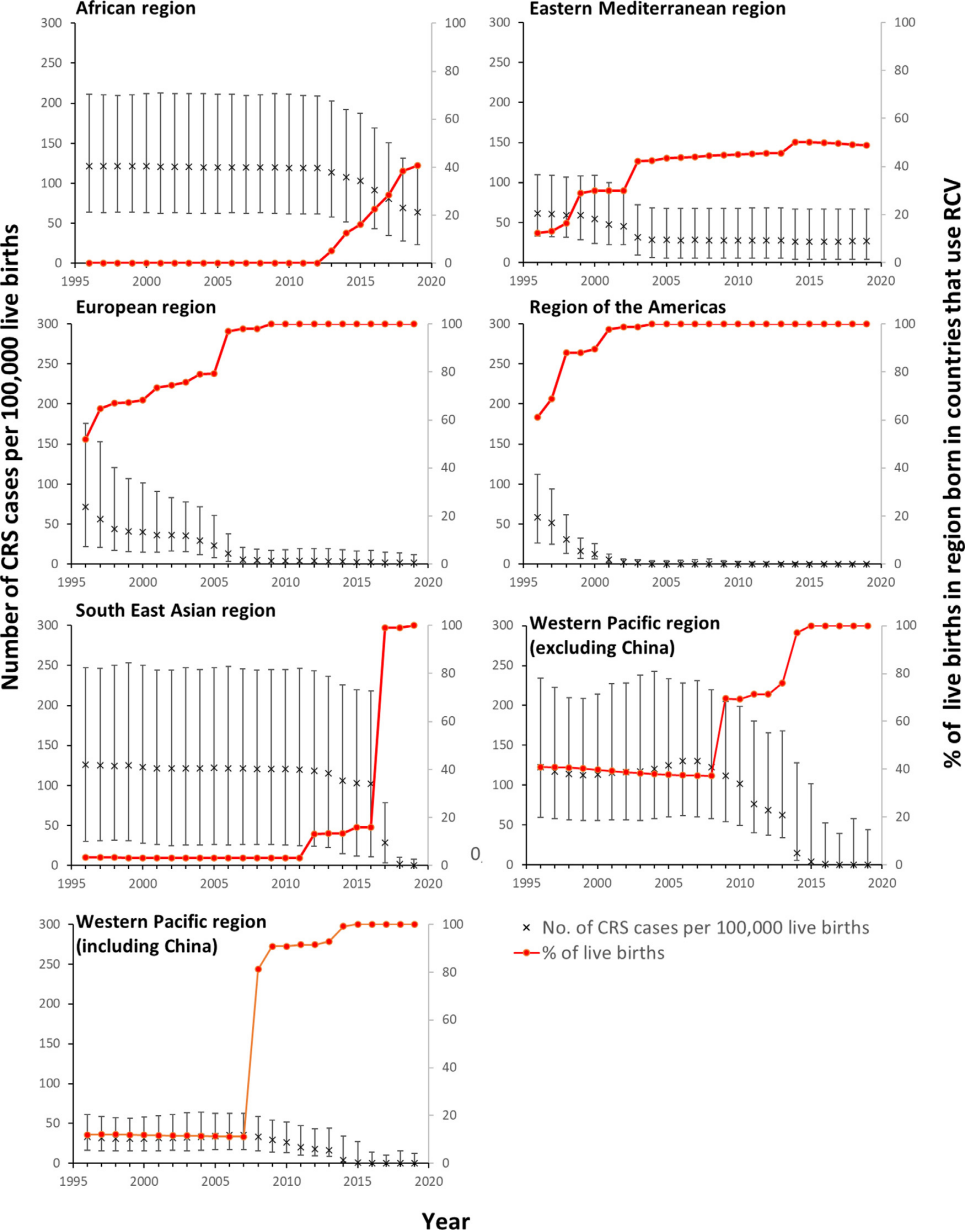
Estimates of the number of CRS cases per 100,000 live births since 1996 for each WHO region and the percentage of live births in each region occurring in countries that had introduced RCV. The error bars show the 95% range (CI). CI, confidence interval; CRS, Congenital Rubella Syndrome; RCV, rubella-containing vaccination.

For 2019, the highest regional CRS incidence (64 (95% CI: 24-123) per 100,000 live births) was estimated for AFR, followed by EMR (27 (95% CI: 4-67) per 100,000 live births) (Figure 2). The estimated CRS incidence was very low elsewhere, for example, <1 (95% CI: <1-8) and <1 (95% CI: <1-12) per 100,000 live births in SEAR and WPR respectively. Almost all the live births in the regions with a low CRS incidence occurred in countries that had introduced RCV (Figure 2).

For AFR, SEAR, and WPR, large declines in the CRS incidence were estimated after 2010 (Figure 2). These declines corresponded to increases in the percentage of the regional live births occurring in countries that had introduced RCV. For AFR, the CRS incidence was stable until 2012, before decreasing from 119 (95% CI: 62-209) to 64 (95% CI: 24-123) per 100,000 live births by 2019. The incidence dropped dramatically in SEAR from 102 (95% CI: 11-218) to <1 (95% CI: <1-8) per 100,000 live births during 2016-2019, having been stable before 2016, with similar reductions in WPR, where it decreased slightly from 2008 and more dramatically from 2013. There has been little change in EMR since 2003. By 2019, 40% and 50% of the estimated live births in AFR and EMR respectively were in countries that had introduced RCV, as compared with 100% in other regions.

**Figure 3:**
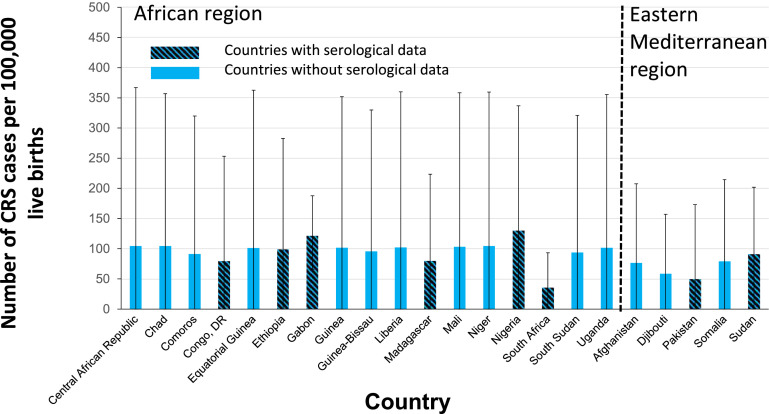
Estimates of the number of CRS cases per 100,000 live births in 2019 for countries which had not yet introduced RCV by mid-2019. The error bars show the 95% range (CI). The patterned and unpatterned bars reflect estimates for countries that had and did not have seroprevalence data respectively. CI, confidence interval; CRS, Congenital Rubella Syndrome; RCV, rubella-containing vaccination.

The 22 countries that had not yet introduced RCV by mid-2019 [19] were in AFR (17, 17% of global live births) and EMR (5, 7% of global live births), representing 60% and 50% of the live births in each respective region. Figure 3 shows the estimated CRS incidence in 2019 in these countries. For the 17 countries in AFR, the estimated CRS incidence was around 100 per 100,000 live births, ranging from 36 (95% CI: 2-93) per 100,000 live births in South Africa to 130 (95% CI: <1 to 337) per 100,000 livebirths in Nigeria. For the five EMR countries, it ranged from 50 (95% CI: <1 to 173) to 91 (95% CI: <1 to 202) per 100,000 live births in Pakistan and Sudan respectively. The CRS incidence in the remaining countries that had introduced RCV remained low (<50 per 100,000 live births) (Supplement Figure S4).

### Number of CRS cases

**Figure 4:**
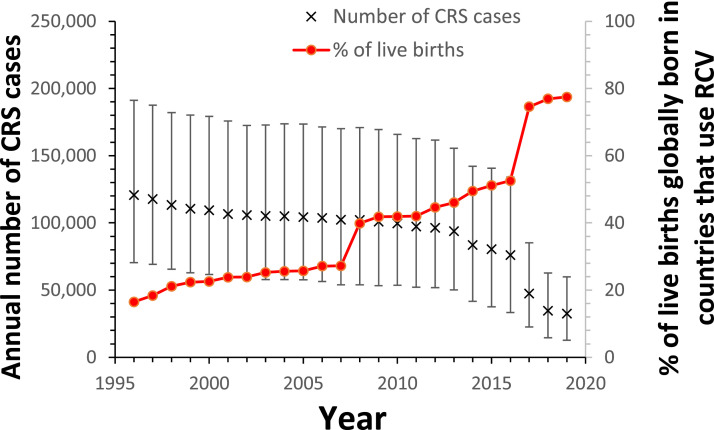
Estimates of the global number of CRS cases born annually during 1996-2019 and the percentage of the live births occurring globally in countries that had introduced RCV. The error bars show the 95% range (CI). CI, confidence interval; CRS, Congenital Rubella Syndrome; RCV, rubella-containing vaccination.

The estimated global annual number of CRS cases declined gradually during 1996-2016 from 121,000 (95% CI: 70,000-191,000) in 1996, reaching 100,000 (95% CI: 54,000-166,000) and 76,000 (95% CI: 33,000-134,000) by 2010 and 2016 respectively (Figure 4 and Supplement Table 13). The estimated number fell more sharply since then, reaching 32,000 (95% CI: 13,000-60,000) by 2019.

By 2019, the highest number of CRS cases were estimated for AFR (25,000 [95% CI: 9200-49,000]), followed by EMR (5700 [95% CI: 870-14,000]) and SEAR (51 [95% CI: <1 to 1660]). The numbers elsewhere were <1 (95% CI: 0-90), 100 (95% CI: <1 to 960), and <1 (95% CI: 0-3770) (AMR, Europe [EUR], and WPR respectively).

Trends in annual number of CRS cases until 2019 differed between regions (Supplement Figure S5), reflecting differences in the population growth rate and timing of RCV introduction. In AFR, secular increases in the number of births, as noted previously [2], were estimated to lead to an increase in the number of CRS cases from 1996, from 29,000 CRS cases (95% CI: 15,000-53,000) to reach 39,000 (95% CI: 20,000-70,000) by 2012, before returning to a similar level to that estimated for 1996 by 2019. In EMR, the estimated annual number of CRS cases changed little after 2004, while in SEAR, it remained relatively unchanged until 2016, and subsequently declined rapidly.

By 2019, the highest average number of CRS cases (>1000 annually) was estimated in seven countries: Democratic Republic of the Congo, Ethiopia, Niger, Nigeria, Uganda, Pakistan, Sudan (Supplement Figure S6). For 11 countries in AFR (Benin, Central African Republic, Chad, Guinea, Liberia, Madagascar, Mali, South Africa, South Sudan) and two countries in EMR (Afghanistan, Somalia), the estimated average annual number of CRS cases was in the range 100-999. For several countries, these estimates had very wide 95% CI, for example, <1 (95% CI: <1 to 3600) for the Philippines.

### Number of CRS cases averted by the introduction of RCV since 2010

Without the introduction of RCV in 2010, we estimate that the global CRS burden would have reached 96,000 (95% CI: 54,000-157,000) by 2019 (Supplement Table 14 and Supplement Figure S7). The corresponding number in AFR and SEAR were similar (44,000 (95% CI: 22,000-79,000) and 40,000 (95% CI: 8700-81,000) respectively); those in EMR and WPR were lower (6,000 (95% CI: 1200-14,000) and 5800 (95% CI: 3400-11,000) respectively) (Supplement Table 14 and Supplement Figure S7). Introducing RCV after 2010 averted an estimated 229,000 (95% CI: 131,000-368,000) CRS cases globally during 2011-2019, with the greatest number averted in SEAR (125,000 95% CI: 37,000-235,000) followed by 65,000 (95% CI: 37,000-111,000), 36,000 (95% CI: 21,000-59,000) and 1500 (95% CI: 400-3300) in AFR, WPR and EMR respectively (Supplement Table 14).

## Discussion

We estimate that the number of CRS cases globally since 2010 has decreased by about two-thirds, from 100,000 (95% CI: 54,000-166,000) to reach 32,000 (95% CI: 13,000-60,000) by 2019, with wide and overlapping confidence intervals. This reduction is largely due to rapid increases in population immunity after introducing RCV, resulting from the WHO preferred strategy to introduce RCV with an SIA covering a wide age range [3]. We estimated dramatic (>90%) reductions in less than a 5-year period in SEAR and WPR, where, by 2019, 100% of births occurred in countries with RCV. Modest reductions were estimated for AFR and EMR, where 40 and 50% respectively of all births were estimated to occur in countries that had introduced RCV by 2019. RCV introduction after 2010 averted an estimated 229,000 (95% CI: 131,000-368,000) CRS cases globally during 2011-2019.

Most of the estimated burden in 2010 was in AFR and SEAR. We estimate that the burden in SEAR has reduced dramatically with the introduction of RCV including widespread SIAs in several populous countries, including Indonesia in 2017 and India in 2018. Similarly, the CRS burden declined in WPR in 2014 after RCV was introduced in Cambodia and Vietnam.

In contrast, the burden in AFR is estimated to have decreased only gradually, after RCV was introduced in less populous countries, such as Senegal (2012), followed by Ghana and Rwanda (2013), Burkina Faso and Tanzania (2014), Cameroon (2015), Eswatini, Gambia, Kenya, Namibia and Zambia (2016). The steady decline could continue, with RCV being introduced in Uganda late in 2019, which was not accounted for in these analyses. Currently, the RCV introductions into the remaining—including the most populous—countries in AFR remain unscheduled. The CRS burden in EMR may change rapidly in the future, since Pakistan introduced RCV through one of the world's largest SIAs in late 2021 [20] and other countries are finalizing plans.

Our transmission model estimated rapid (>50%) reductions in CRS incidence soon after rubella vaccination is introduced. Such rapid reductions are consistent with observed data and with findings from other models investigating the introduction of RCV with SIAs [21]. For example, a recent study [22] in five African countries, in which RCV was introduced through an SIA targeting children aged 9 months to 14 years in 2013 found a typical 48-96% reduction in the mean incidence of confirmed rubella cases after the SIA, compared to that beforehand.

Our estimates are subject to several limitations, as detailed elsewhere [2], including factors relating to the representativeness of the population surveyed in the seroprevalence data. For example, many datasets were convenience samples which may not be representative of the general population, and many countries lacked seroprevalence data so that, for those countries, data from countries in the same WHO region were used instead. For simplicity, we have not included the effect of correlations between vaccine doses and between vaccination received routinely and those received in a SIA. The correlation between recipients of vaccine doses may be population-specific. For example, in one recent study, the percentage of children who had not received measles vaccination who subsequently received vaccination during an SIA was inversely affected by wealth status [23]. By assuming that all children are equally likely to receive a second vaccine dose, irrespective of whether they have received the first vaccine dose, we may have overestimated the reduction in transmission and potentially the CRS incidence as a result of the introduction of RCV in some settings.

In these analyses, we have used the actual vaccine coverage and specific ages reported for each SIA. In many countries, campaigns that were used to introduce RCV in the population were reported for individuals in the age range 9 months to 14 years, for which GAVI the Vaccine Alliance provided financial support during the past decade. Residual immunity gaps may have remained; therefore, sporadic rubella transmission affecting women of child-bearing age and CRS cases could have continued to occur.

We may have overestimated the CRS burden in some settings by only including vaccination occurring through the routine schedule or during SIAs, given recent WHO recommendations [24] to give one dose of RCV to all unvaccinated or seronegative non-pregnant women, who are potentially identified at pre-marital screening, post-partum or when they contact the health system for other reasons. Conversely, we may have underestimated the impact of vaccinating children on transmission among adults by basing assumptions relating to age-dependent mixing patterns on studies from Western settings, where, compared to adults in low and middle-income settings, adults may be less likely to mix with children [25].

Our results rely on estimates of the pre-vaccination force of infection based on seroprevalence data from populations in which routine RCV vaccination had not been introduced. With the introduction of RCV in many countries, new studies collecting such data are increasingly rare. For example, our systematic review, which considered the same databases and search criteria as those used previously, identified fewer than 20 seroprevalence studies over the period 2012-2020 that were eligible for inclusion. However, as illustrated in previous analyses [14], a country with a recent RCV introduction can collect seroprevalence data in unvaccinated age groups, from which we can estimate the pre-vaccination force of infection after we make assumptions about the reduction in the force of infection resulting from vaccination.

Future trends in the CRS burden depend on many factors, including importation and variations in coverage. For simplicity the effects of importation of infection have not been included here and, as for measles and other infections [26], such importations could potentially lead to outbreaks, with the extent of sustained transmission depending on the population's immunity levels. Variations in coverage, resulting from the ongoing COVID pandemic may have also affected the CRS burden. WUENIC data indicate that the global coverage of RCV decreased from 69% to 66% [27]. Disruptions and delays to routine vaccination and the implementation of SIAs could cause increases in the proportion of women reaching child-bearing age still susceptible, which, coupled with ongoing transmission, could potentially lead to eventual increases in the CRS burden.

In conclusion, between 2010 and 2019, we estimate a reduction of over two-thirds in the global CRS burden, with 229,000 (95% CI: 131,000-368,000) cases averted during 2011-2019, following the updated WHO introduction strategy, with a catch-up SIA and resources available through GAVI-funded initiatives. Our model estimated particularly dramatic (>90%) reductions in CRS incidence in SEAR and WPR in less than 5 years. With sustained coverage and the introduction of RCV into the remaining countries where it has not yet been introduced, further reductions are possible, making the long-term goal of eradication feasible. These hard-won achievements must not be destabilized by the consequences of the COVID pandemic.

## Declaration of competing interests

The authors have no competing interests to declare.

## Appendix Supplementary materials

Image, application 1
