# Supplementary material for: Estimates of the global burden of Congenital Rubella Syndrome, 1996-2019
Source: Int J Infect Dis. 2023 Dec;137:149–56. doi: 10.1016/j.ijid.2023.09.003 (PMC10689248; doi:10.1016/j.ijid.2023.09.003)
Supplement: Supplementary file 1 [file mmc1.pdf]

Estimates of the global burden of Congenital Rubella Syndrome, 1996-2019

Supplementary Material

Table of contents

A. Vaccination coverage data……………………………………………………………..………4

Table 1: Countries and time periods for which RCV coverage for a given dose was estimated. One of four approaches was used: linear interpolation, constant gap, flat line and set value approaches.……………………………………………………………………………..5

B. Analyses of additional seroprevalence datasets…………………………………….....6

C. Overview of the transmission model

Figure S1: General structure of the transmission model in the absence of vaccination...7

Table 2**:** Summary of the definitions of compartments and variables used in the model. Where necessary in the equations, the subscript "w" is used to denote females...9

Table 3**:** Summary of the definitions of the transition-related and other parameters ……….5

D. Values of parameters that were varied in the transmission model…………………………..…………………………………………………….14

Table 4: Summary of the basecase and ranges of the parameters used in the transmission model..14

Table 5: Characteristics of the three studies used in these analyses for defining the risk of a child being born with CRS if the mother is infected with rubella during the first 16 weeks of pregnancy……………………………………………………………………………………...14

Table 6: Data from the three studies[33-35] on the risk of congenital rubella infection, risk of probable CRS given congenital rubella infection and the risk of probable CRS following rubella infection in the mother during the first 16 weeks of pregnancy………………………………………………………………………………………17

Table 7: Data from the three studies that were used to generate the distribution of the risk of probable CRS given maternal rubella infection during the first 16 weeks of pregnancy………………………………………………………………………………………18

Table 8: Estimated risk of a child being born with probable CRS following rubella infection in the first 16 (Miller et al and Grillner et al) or 18 (Hahne et al) weeks of pregnancy…………………………………………………………………………….……..20

Figure S3: Distribution of the bootstrap replicates of the risk of a child being born with probable CRS following rubella infection in the mother during the first 16 (Miller et al and

Grillner et al) or 18 (Hahne et al) weeks of pregnancy. See Table 6, Table 7 and Table 8 for further details about the assumptions for Grillner et al and Hahne et al. The top left figure also shows the outcome of taking 1000 random samples from the Gamma distribution with shape and scale parameters 37 and 56 respectively..20

Table 9: Method for compiling 1000 values for the pre-vaccination force of infection for a given country according to the number of seroprevalence datasets available for that country. Further details of the actual datasets used are in Table 10 and Table 11................................21

Table 10: Datasets used to set up 1000 force of infection bootstrap files for the WHO Regions. These bootstrap files were used to generate 1000 contact parameters for use in the transmission model to calculate the median and 95% range of the CRS burden for countries in a given region which did not have any pre-vaccination seroprevalence data (see main text). Note that these datasets had been accepted after performing the selection procedure described in section B. ………...22

Table 11: Summary of the bootstrap datasets used to establish the pre-vaccination force of infection for each country using the transmission model, using the WHO regional grouping to assign datasets for countries without serological datasets from before the introduction of RCV. See Table 10 for the datasets used to make up the bootstrap datasets..23

E: Equations for the CRS incidence and annual numbers of CRS cases……….……..31

F. Estimates of the pre-vaccination force of infection………..……………………………33

Table 12: Summary of the additional datasets that were identified since the previous systematic review, best-fitting values for the force of infection and (where appropriate) the sensitivity of the antibody assay for each catalytic model before the introduction of RCV. The values in parentheses reflect the 95% confidence intervals, obtained by bootstrapping. Several of the estimates were published in an interim update of the literature review[28] and are included here for completeness.…………………………………………………..…….34

G. Estimates of the CRS incidence……………………………………………………….47

Figure S4: Average estimates of the number of CRS cases per 100,000 live births in 2019 for all countries.………………………………………………………...…………………47

Table 13: The median CRS incidence per 100,000 live births and number of CRS cases born in each WHO region and worldwide in 1996, 2000, 2010 and 2019 and the percentage of the regional live births occurring in countries which had introduced RCV by these years. The numbers in parentheses reflect 95% confidence limits...……………………………..………..48

Figure S5: Estimates of the number of CRS cases born annually in each WHO region during 1996-2019. The error bars show the 95% range (CI)..49

Figure S6: Estimates of the average number of CRS cases born annually in each country in 2019……………………………………………………………..…………………………50

Table 14: Comparison between the estimated number of CRS cases in 2010, 2019 and during 2011-2019 with vaccination as implemented, and the numbers that might have been expected if vaccination had not been introduced after 2010. The final column shows the

estimated number of CRS cases averted during 2011-2019 by the introduction of RCV after 2010..51

Figure S7: Estimates of the annual number of CRS cases during 1996-2019 globally, in the African, Eastern Mediterranean, South East Asian regions, as calculated using the RCV coverage as implemented and the number that might have been seen if there had been no new introductions of RCV after 2010. The shaded areas show the 95% confidence intervals.…………………………………………………………………………….………..52

A. Vaccination coverage data

The vaccination coverage data that were used in the model are described in the main text. Missing SIA or routine coverage data were further supplemented from publications[1-22]. Data on coverage among adolescent girls came from publications where possible[23-25] (see main text for details).

Table 1: Countries and time periods for which RCV coverage for a given dose was estimated. One of four approaches was used: linear interpolation, constant gap, flat line and set value approaches.

Linear interpolation

ISO	Dose	Years
AND	1st	1989-1996
AND	2nd	1991-1996
ARG	2nd	1998-1999
ATG	2nd	1997-2004
AUT	2nd	1994-1999
BHS	2nd	2001-2003
BRA	1st	1992-1999
CAN	2nd	2003,2005,2007,2008,2010
DEU	1st	1988-1990
GRC	2nd	1999-2006
IRL	2nd	1995-1997
ISL	F	1982
JPN	2nd	2006-2007
KOR	2nd	1997-1999
LBN	2nd	1996-1999
MHL	1st	1989
SGP	F	1977-1980
SVN	2nd	1999

Fixed gap[%]

ISO	Dose	Years	Source year(s) of gap estimate
AND	2nd	1997-2006	2007
ARE	2nd	1995-1999	2000
BEL	2nd	1994-2005	2006
BRN	2nd	1996-1999	2000
BTN	2nd	2006-2007	2008
CHE	2nd	1997-2004	2005
CHL	2nd	1992-2003	2004-2018*
COL	2nd	1997-1999	2000
CPV	2nd	2016	2017
CRI	2nd	1992-2003	2008-2018*
CYP	2nd	1999-2006	2007-2018*
CZE	2nd	1995-1999	2000
ESP	2nd	1995-2003	2004
EST	2nd	1994-1999	2000
FIN	2nd	1982-2015	2016-2018*
FRA	2nd	1996-2009	2010
GBR	2nd	1996-1999	2000
GRD	2nd	2000-2001	2002-2018*
HRV	2nd	1994-2000	2001
ISL	2nd	1987-2002	2003-2010*
LUX	2nd	1995-2012	2013
MEX	2nd	1998-1999	2000-2018*
MNE	2nd	1996-1999	See Serbia dose 2
NZL	2nd	1992-2010	2011
PRT	2nd	1990-1999	2000
QAT	2nd	1992-2001	2002
RUS	2nd	1997-1999	2000
SLV	2nd	2000-2002	2003
SRB	2nd	1996-1999	2000
SVK	2nd	1994-1999	2001
SVN	2nd	1992-1998	2000
SWE	2nd	1982-1999	2000-2018*
TON	2nd	2002	2003

Flat line

ISO	Dose	Years	Source year(s)
AUS	F	1989-1998	1988
AUS	M	1993-1998	1988
BRN	F	1988-1998	1987
CYP	F[#]	1972-1979	1980
DEU	2nd	1986-1990	14%[$]
DEU	2nd	1992-1997	1998
HKG	F	1978-1979	1980
IRL	F	1980-1982	1983
IRL	2nd	2000-2011	Mid-point for 1999 & 2012
IRL	2nd	2013-2018	2012
KAZ	F	2007-2009	2007
LBY	1st	1993-2000	2001 (MCV2)
MCO	2nd	1996-2013	2014
MYS	F	2002-2009	2001
MYS	3rd[&]	2016-2023	2015
NZL	F	1990-1991	1989
NZL	F	1979	1980
PRT	F	1987-1989	1986
SGP	2nd	1982-1998	1981

Set value

ISO	Dose	Years	Assumed value	Reasoning
CPV	1st	2010	25%	Introduced in Sept 2010
JPN	F	1977-1981, 1996	70%	Consistent with constant (67-77%) coverage during 1982-1994
SGP	M	1982-1990	100%	Mandatory conscription- Vx likely.

[%] The coverage is calculated so that the absolute difference in the coverage between the first and second doses in the given year is fixed at the level observed in the years in column 7, unless otherwise stated. Consistent with steady coverage of the first dose.

[#] Adolescent

[$] Assumed to be at the level for the whole of Germany in 1990, multiplied by 0.797 (the factor by which the population in W Germany differed from the total population in Germany

* Calculated as the average annual difference between the first and second dose coverage for the period specified

[&] Provided for 7 year olds

B. Analyses of additional seroprevalence datasets

The methods used to estimate the force of infection for the seroprevalence datasets identified since the previous related analyses are described in [26]. In brief, four catalytic models (A, B, C, D) were fitted to the age-stratified seroprevalence data to estimate the average annual "force of infection" among <13 and ≥13 year olds (i.e. the rate at which susceptible <13 and ≥13 year olds are infected), and the sensitivity of the antibody assay. The pre-vaccination force of infection was allowed to either differ for those aged <13 and ≥13 years (models A and B) or be identical for all ages (models C and D). The sensitivity of the rubella serological (antibody) assay was either estimated (models A and C) or assumed to be 100% (models B and D).

The criteria for selecting the force of infection for further use are described in [26]. In brief, the force of infection estimates were selected in decreasing order of biological plausibility of the model, coming from model A, unless they met specific criteria (the force of infection was implausibly high (>600 per 1000 per year), zero in either age group, higher for older individuals than for children or its upper confidence limit was 100%). If this occurred, we used estimates from model B in preference to those from model C, and those from model C in preference to those from model D. If no model fitted the data convincingly, occurring when the best-fitting age-specific proportion seronegative passed through the 95% confidence limits of just one of the observed datapoints, the dataset was dropped from further analyses.

We included an additional criterion for countries for which the sensitivity of the assay was known to be high that model B was selected in preference to model A if all the other criteria were satisfied and the estimated sensitivity of the assay was 100%for model A, and the lower limit of the 95% confidence interval was implausibly low (less than 95%)[27].

C. Overview of the transmission model

General structure and equations

The transmission model used is based on the one used in previous calculations of the global burden of CRS. We summarise the key features below; further details can be found elsewhere[26, 28].

Figure S1 summarises the general structure of the model in the absence of vaccination. The population is stratified into those who have maternal immunity, those who are susceptible, pre-infectious (infected but not yet infectious), infectious and immune. The demography in the model was described using a realistic age structured (RAS) population following Schenzle's approach[29], with individuals stratified by sex and into 99 age strata, corresponding to the ages <1, 1-<2, 2-<3, …, <99 years.

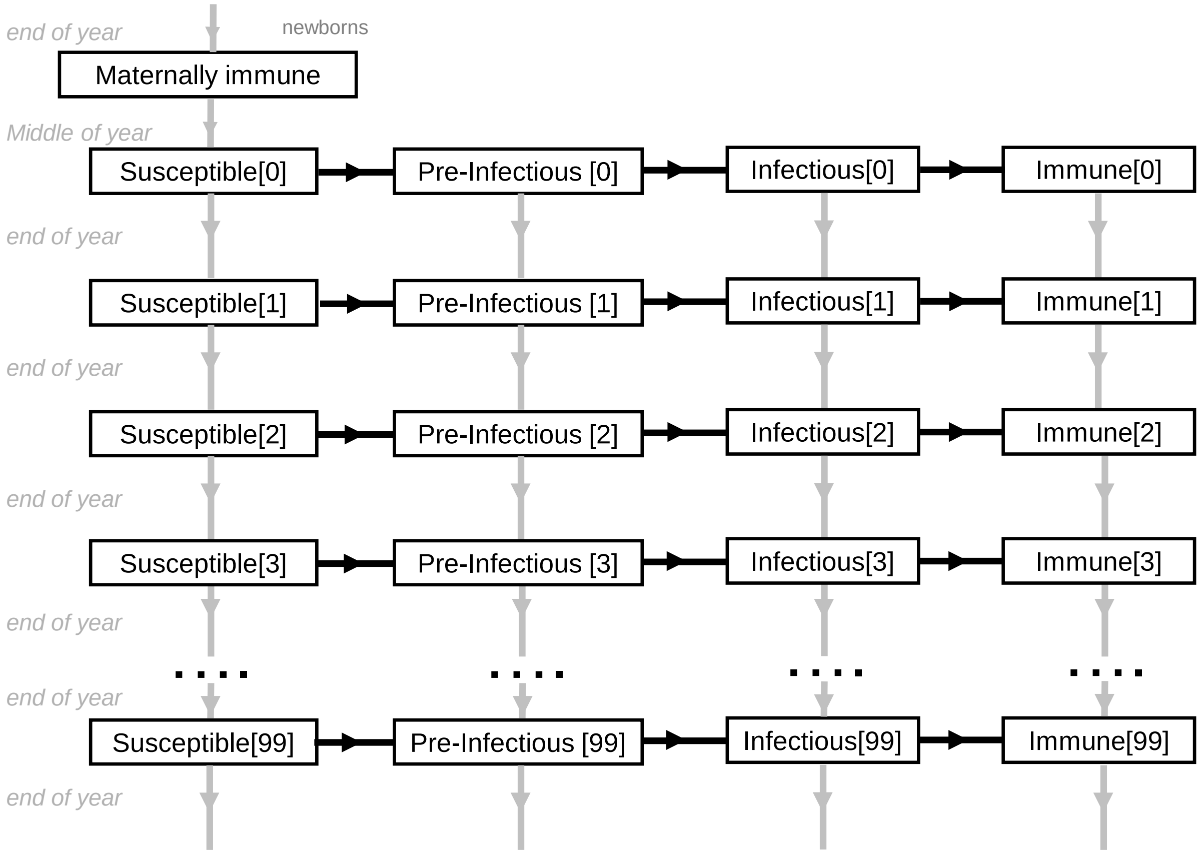

Figure S1: General structure of the transmission model in the absence of vaccination.

For all countries, we used the country-and age-specific mortality rate, calculated from survival data for the period 2015-2020 from UN population databases[30]. Therefore, the number of people of age *a* at a given time *t* $N_a(t)$ depends on the mortality rate. The number of births in the model was calculated by multiplying model predictions of the population size in the given year by the crude annual per capita birth rate for the period 2015-2020, obtained from UN population databases[30]. Both the mortality and crude birth rates in the model were assumed to be fixed over time. Note that the absolute magnitude of the numbers of births in the model does not greatly influence the absolute numbers of CRS cases predicted for the global burden, since these were calculated by multiplying model predictions of the age-specific number of CRS cases per live birth by the observed numbers of live births by maternal age, as seen in UN population databases[30] and then summing the resulting numbers over all maternal ages.

Individuals are born into the first age stratum (stratum *a*=0) on the 31st August of each year and are assumed to have maternal immunity for 6 months. Following standard approaches[29] individuals in each age stratum move to the subsequent age stratum on the 31st August of each year, at the same time as vaccination occurs (see below), and leave the model once they reach age 100 years.

The force of infection in the model at a given time *t* ($\lambda_y(t)$ and $\lambda_o(t)$) depends on age-specific contact between people and the prevalence of infectious people, with the contact parameters calculated from average annual force of infection estimates from seroprevalence data (see below). For convenience, vaccination is implemented on a single day each year in the model, except for children that are vaccinated when aged 9 months (see below).

Table 2 and Table 3 give definitions of the variables and parameters respectively that are used in the model. Throughout the description, we use the subscript "*y*" to refer to younger

individuals (aged <13 years) and the subscript "*o*" to refer to older individuals (aged ≥13 years). Where necessary, the subscript "w" is used to denote females.

Table 2**:** Summary of the definitions of compartments and variables used in the model. Where necessary in the equations, the subscript "w" is used to denote females.

**Variable**	**Definition**
$M_g(t)$	Number of individuals of gender *g* with maternal immunity at time *t*.
$S_{a,g}(t)$	Number of susceptible individuals of gender *g* aged *a* years at time *t.*
$E_{a,g}(t)$	Number of individuals in the pre-infectious category (infected but not infectious) of gender *g* and age *a* years at time *t.*
$I_{a,g}(t)$	Number of infectious individuals of gender *g* and aged *a* years at time *t.*
$I_y(t)$, $I_o(t)$	Number of younger and older infectious individuals at time *t.*
$\bar{I}_y$, $\bar{I}_o$	Average number of younger and older infectious individuals before the introduction of vaccination.
$R_{a,g}(t)$	Number of individuals of gender *g* and aged *a* years at time *t* who are immune either as a result of vaccination or natural infection.
$N_a(t)$	Total number of people (males and females combined) aged *a* at time *t*.
$N_y(t)$, $N_o(t)$	Total number of younger and older individuals at time *t* (aged <13 and ≥13 years respectively).

Table 3**:** Summary of the definitions of the transition-related and other parameters

**Parameter**	**Definition**
$\lambda_a(t)$, $\lambda_y(t)$, $\lambda_o(t)$	The force of infection for individuals in a given age group at time *t*. The subscript *a* refers to people of age *a;* the subscripts *y* ('younger') and *o* ('older') refer to individuals aged <13 and ≥13 years respectively.
$\overline{\lambda}_y$, $\overline{\lambda}_0$	The average force of infection before the introduction of vaccination for individuals aged <13 years ('younger') and ≥13 years ('older'), respectively.
β_{yo}	The rate at which specific younger susceptible individuals come into effective contact with older infectious persons per unit time. An effective contact is defined as one which is sufficient to lead to transmission between an infectious and susceptible individual[31]. The definitions of β_{oy}, β_{oo}, β_{yy} are analogous.
c_{yo}	The number of younger susceptible individuals effectively contacted by each older infectious person per unit time. An effective contact is defined as one which is sufficient to lead to transmission between an infectious and susceptible individual[31]. The definitions of c_{oy}, c_{oo}, c_{yy} are analogous.
$m_{a,g}$	The mortality rate for individuals of age *a,* gender *g*. The rate is calculated using survival data for 2015-2020 from UN population databases. [30]
$v_{a,g}(t)$	The proportion of individuals of age *a* of gender *g* who are vaccinated at time *t*. The coverage data are those estimated and/or reported to WHO and supplemented by the literature, where available.
v_e	The vaccine efficacy. This is varied between 85% and 99%, with a median of 95% (see Table 4) and accompanying text.
$B_g(t)$	The numbers of live births was calculated as the product of the predicted population size and the crude per capita birth rate, obtained from UN population databases [30]. Note that the absolute magnitude of the numbers of births in the model does not greatly influence the absolute numbers of CRS cases predicted for the global burden, since these were calculated by multiplying model predictions of the age-specific number of CRS cases per live birth by the observed numbers of live births by maternal age, as seen in UN population databases [30] and then summing the resulting numbers over all maternal ages.
f	The rate at which individuals in the pre-infectious category become infectious, taken to equal 0.1/day, equivalent to assuming an average pre-infectious period of 10 days.
r	The rate at which infectious individuals recover and become immune, taken to equal 0.909 per day, equivalent to assuming an average infectious period of 11 days.
r_{CRS}	Risk that a child is born with CRS following rubella infection in the mother during the first 16 weeks of pregnancy. Assumed to be 65%, with a 95% range of 47-88%. See Table 4 and the accompanying text for further details.
a_f	Oldest age group in the model (99 years)
T_{crs}	Time period (16 weeks) following the start of pregnancy during which there is an increased risk of the child being born with CRS, if the mother is infected whilst pregnant.
T_E	Last year of the model simulations, 2019

The differential equations in age stratum *a* (a=0, 1, 2, ...,99 years) are provided below (see Table 2 and Table 3 for the definitions of variables and parameters).

$$\frac{dM_g(t)}{dt} = -m_{0,g}M_g(t) \qquad\qquad 0 < t < 182\, mod\, 365$$

$$\frac{dS_{a,g}(t)}{dt} = -\lambda_a(t)S_{a,g}(t) - m_{a,g}S_{a,g}(t)$$

$$\frac{dE_{a,g}(t)}{dt} = \lambda_a(t)S_{a,g}(t) - m_{a,g}E_{a,g}(t) - fE_{a,g}(t)$$

$$\frac{dI_{a,g}(t)}{dt} = fE_{a,g}(t) - m_{a,g}I_{a,g}(t) - rI_{a,g}(t)$$

$$\frac{dR_{a,g}(t)}{dt} = rI_{a,g}(t) - m_{a,g}R_{a,g}(t)$$

The equations for the transitions occurring on 31st August each year are as follows:

$M_g(t) = B_g(t)$

$S_{a,g}(t) = S_{a-1,g}(t-\delta t)(1- v_e\, v_{a,g}(t) - m_{a-1,g} - \lambda_{a-1}(t-\delta t))$ for $0< a \leq a_f$ years

$E_{a,g}(t) = E_{a-1,g}(t-\delta t)(1-m_{a-1,g} - f)+\lambda_{a-1}(t-\delta t)S_{a-1,g}(t-\delta t)$ for $0< a \leq a_f$ years

$I_{a,g}(t) = I_{a-1,g}(t-\delta t)(1-m_{a-1,g}-r)+f\, E_{a-1,g}(t-\delta t)$ for $0< a \leq a_f$ years

$R_{a,g}(t) = R_{a-1,g}(t-\delta t)(1-m_{a-1,g}-r)+rI_{a-1,g}(t-\delta t)+ v_{a,g}(t)v_e\, S_{a-1,g}(t-\delta t)$ for $0< a \leq a_f$ years

The equations for the transitions occurring 6 months after the 31st August (or equivalently, 28th February), when individuals in the first year of life lose their maternal immunity are:

$S_{0,g}(t) = M_g(t-\delta t)(1-m_{0,g})$

$M_g(T) = 0$

Vaccination for children in their first year of life that is scheduled before 12 months of age is implemented at age 9 months, using the following equations:

$S_{a,g}(t) = S_{a,g}(t-\delta t)(1- v_e\, v_{a,g}(t) - m_{a,g} - \lambda_a(t-\delta t))$ for $a=0$ years

$R_{a,g}(t) = R_{a,g}(t-\delta t)(1-m_{a,g}-r)+rI_{a,g}(t-\delta t)+ v_{a,g}(t)v_e\, S_{a,g}(t-\delta t)$ for $0< a \leq a_f$ years

The equations were solved using a specially written C-program, using the Euler method with a time step, δt, of 0.25 day. The model was run for 170 simulated years before the earliest possible introduction of RCV (1970), starting from the equilibrium numbers of individuals in each compartment, and with a population size of 750,000 ($N(T_0)$), with equal numbers of males and females.

Describing transmission between people in the model

The contact parameters in the model were assumed to differ between younger and older individuals according to the following matrix of "Who Acquired Infection From Whom":

$$\begin{pmatrix} \beta_1 & 0.7\beta_2 \\ 0.7\beta_2 & \beta_2 \end{pmatrix}$$

The contact parameters in the model for each country were calculated for each bootstrap estimate for the force of infection for younger and older individuals, before the introduction of vaccination using methods described previously[26]. For a given assumption about contact between individuals, the force of infection at time t for individuals among younger and older individuals ($\lambda_y(t)$ and $\lambda_o(t)$ respectively), is given by the following equations:

$$\lambda_y(t) = \frac{c_{yy} I_y(t) + c_{yo} I_o(t)}{N_y(t)}$$

$$\lambda_o(t) = \frac{c_{oy} I_y(t) + c_{oo} I_o(t)}{N_o(t)}$$

c_{yy}, c_{yo}, c_{oy} and c_{oo} are related to β_{yy}, β_{yo}, β_{oy} and β_{oo} through the following equations, where T_0 is the start of the model runs:

$$c_{yy} = \beta_{yy} N_y(T_0)$$
$$c_{yo} = \beta_{yo} N_y(T_0)$$
$$c_{oy} = \beta_{oy} N_o(T_0)$$
$$c_{oo} = \beta_{oo} N_o(T_0)$$

The parameters, β_1 and β_2 in the WAIFW matrix for given values for the average force of infection before the introduction of vaccination among younger and older individuals for a given country (denoted by $\bar{\lambda}_y$ and $\bar{\lambda}_o$ respectively) were calculated using the following equations:

$$\beta_1 = \frac{\bar{\lambda}_y - 0.7\beta_2 \bar{I}_o}{\bar{I}_y}$$

$$\beta_2 = \frac{0.7\bar{\lambda}_o}{0.7\bar{I}_y + \bar{I}_o}$$

Where $\bar{I}_y$ and $\bar{I}_o$ are the average numbers of infectious people (males and females combined) for younger and older people, respectively. $\bar{I}_y$ and $\bar{I}_o$ are calculated using the approximations $\bar{I}_y \approx \bar{\lambda}_y \bar{S}_y D$ and $\bar{I}_o \approx \bar{\lambda}_o \bar{S}_o D$, where D is the duration of infectiousness and $\bar{S}_y$ and $\bar{S}_o$ are the average numbers of susceptible children and older individuals respectively, calculated as follows:

$$\bar{S}_y = \sum_{a=1}^{a_y} N_a(T_0) e^{-\bar{\lambda}_y (a-0.5)}$$

$$\bar{S}_o = \sum_{a=a_y+1}^{a_f} N_a(T_0) e^{-12.5\bar{\lambda}_y} e^{-\bar{\lambda}_o (a-a_y)}$$

Here, the number of people of age *a* was calculated using the following equation, namely by multiplying the population size at the start ($N_a(T_0)$) by the proportion of the population at equilibrium that was of age *a:*

$$N_a(T_0) = \sum_{a=a_y+1}^{a_f} N(T_0) \frac{N_a(T_E)}{\sum_{a=0}^{a_f} N_a(T_E)}$$

The equilibrium numbers of people in each age group were obtained by running the model until 2019 in the absence of vaccination.

D. Values of parameters that were varied in the transmission model

Table 4: Summary of the basecase and ranges of the parameters used in the transmission model.

	**Base-case value**	**Values used in sensitivity analyses**	**Basis**
**Pre-vaccination force of infection (used to calculate contact parameters)**	Based on pre-vaccination seroprevalence data from the country (if available) or from the same WHO region otherwise.	1000 bootstrap-derived values	See [26] and main text.
**Vaccine efficacy**	95%	85% to 99%, sampled from the truncated Beta distribution with parameters α=33 and ß=2.	Leads to a median vaccine efficacy of 95%. Consistent with estimates from [32]
**Vaccination coverage**	See main text	10% higher or lower each year than historical projections.	Plausible
**Risk of a child being born with CRS if the mother is infected during the first 16 weeks of pregnancy**	65%	Sampled from the Gamma distribution with shape and scale parameters 37 and 56 respectively.	Lead to a median and 95% range of 65% and 47-88% respectively consistent with those from several studies[33-35]. See below for further details.

Vaccine efficacy

The vaccine efficacy assumed in our analyses is consistent with estimates of the vaccine effectiveness from a recent systematic review and meta analysis[32], which identified four suitable studies for calculating a pooled estimate of the vaccine effectiveness for the most widely-used strain (RA27/3). The pooled estimate was found to be 97% (95% CI: 92-99%) with the estimate from the study with the greatest weight (41.54 vs 22.55, 22.53 and 13.38) in the meta analysis being 95% (95% CI: 84-98%). The beta distribution used in our analyses results in a median vaccine efficacy of 95% (Figure S2), and the range is

consistent with the pooled vaccine effectiveness estimate and the range from the study with the greatest weight.

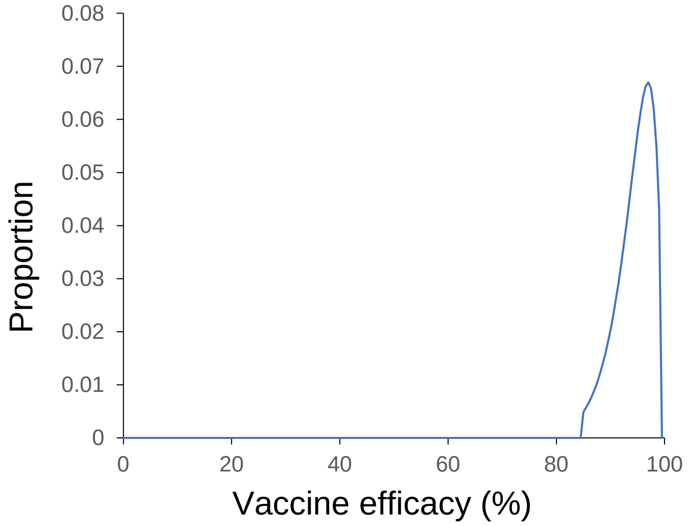

Figure S2: Illustration of the truncated beta distribution with α=33 and ß=2 used to sample values for the vaccine efficacy.

The risk of a child being born with CRS if the mother is infected during the first 16 weeks of pregnancy

A systematic review by Thompson et al[36] identified three studies as being the most reliable for estimating the risk of a child being born with CRS, given rubella infection in the mother during the first 16 weeks of pregnancy. Table 5 summarizes the characteristics of these studies. The study of Miller et al[33] is considered to be the most reliable of these studies, considering 1016 pregnant women with laboratory-confirmed rubella infection in England and Wales during January 1976 – September 1978. The other studies considered 491 (Grillner et al[34]) and 32 (Hahne et al[35]) pregnant women respectively. The percentage of infected women who continued with their pregnancies to term ranged from 42% or 378/966 (Miller et al[33]) to 64% (315/491) and 94% (30/32) in Grillner et al[34] and Hahne et[35] al respectively.

Table 5: Characteristics of the three studies used in these analyses for defining the risk of a child being born with CRS if the mother is infected with rubella during the first 16 weeks of pregnancy

	Miller et al[33]	Grillner et al[34]	Hahne et al[35]
Study population	All pregnant women who had rubella confirmed by a Public Health Laboratory Service laboratory in England and Wales between January 1976 and September 1978	Laboratory-confirmed rubella cases among pregnant women during 1978-1980 in Sweden	Laboratory-confirmed rubella cases among pregnant women during a rubella outbreak during September 2004- July 2005 in The Netherlands and Canada. Population objected to vaccination on religious grounds
Initial number of pregnant women	1016	491	32
% of all pregnancies resulting in livebirths (n/N)	42% (378/966)	64% (315/491)	94% (30/32)
Outcome of pregnancies with rubella infection in the first trimester	6% and approximately 50% of pregnancies with infection during 1st 12 weeks and 13-16 weeks respectively continued	35 livebirths (out of 176 infected women)	14/16 pregnancies resulted in livebirths
Definition of intra-uterine infection	Presence of IgM antibodies soon after birth or persistence of IgG after the first year	Presence of rubella antibodies at age 8 months and/or rubella-specific IgM antibodies at birth	WHO European Region case definitions

All three studies provided estimates of the risk of congenital infection and the risk of a child being born with adverse outcomes relating to rubella infection (referred to here, for simplicity, as probable CRS), given infection in the mother at different stages of pregnancy (Table 6). For a given study, the overall risk of probable CRS given rubella infection in the mother at a given stage can be computed as the product of the risk of congenital infection at that stage and the risk of probable CRS, given congenital rubella infection.

Table 6: Data from the three studies[33-35] on the risk of congenital rubella infection, risk of probable CRS given congenital rubella infection and the risk of probable CRS following rubella infection in the mother during the first 16 weeks of pregnancy

	Stage (weeks) in pregnancy of maternal rubella infection	Duration of the pregnancy stage (weeks)	Risk (%) of a child being congenitally infected with rubella (number seropositive/number tested)	Risk (%) of probable CRS given congenital rubella infection (number with probable CRS/number with congenital infection followed up)	Risk (%) of probable CRS following rubella infection in the mother*
Miller et al	<11	10	90 (9/10)	100 (9/9)	90
	11-12	2	67 (4/6)	50 (2/4)	33
	13-14	2	67 (12/18)	17 (2/12)	11
	15-16	2	47 (17/36)	50 (7/14)	24
Grillner et al	1-8 (assumption 1)**	8	40 (2/5)	100 (2/2)	40
	1-8 (assumption 2)**	8	66% (2/3)	100 (2/2)	66
	9-12	4	57 (4/7)	75 (3/4)	43
	13-14	2	57 (4/7)	50 (2/4)	29
	15-16	2	70 (7/10)	14 (1/7)	10
Hahne et al***	4-10	7	90 (9/10)	100 (9/9)	90
	11-12	2	50 (2/4)	100 (2/2)	50
	14-18	5	33 (2/6)	0 (0/2)	0

* Calculated as the product of the risks in columns 4 and 5 for all periods.
** Grillner et al mention that two mothers may have been infected with rubella before conception. If this occurred, two children would have been at reduced risk of rubella infection from the mother, compared to children infected at other stages of pregnancy. When calculating the risk that the children were infected, these two children are included in the denominator for assumption 1 and are excluded for assumption 2.
***The data for Hahne et al are taken from Table 1 in the paper, which includes infections in both The Netherlands and Canada, which were part of the same outbreak

For each study, as elsewhere[26, 37], we calculated the risk of probable CRS given infection in the mother during the first 16 weeks of pregnancy as the weighted average of the risk of probable CRS given infection in the mother during the different stages for the first 16 weeks of pregnancy. We used bootstrapping to compute 95% confidence intervals and distribution of the risk of probable CRS given rubella infection in the mother during the first 16 weeks of pregnancy for each study, as described below.

For each period of infection *g* in the study, we used the risk of probable CRS given infection in the mother at that time (r_g) to generate 10,000 bootstrap samples for the number of children who were born with probable CRS, denoted as $c_{i,g}$ for the ith bootstrap replicate.

For a given bootstrap *i*, we used the following equation to compute the weighted average of the risk (w_i) that a child was born with an outcome consistent with CRS following infection in the mother during the first 16 weeks of pregnancy:

$$w_i = \sum_{g=1}^{G} \frac{c_{i,g}}{n_g} d_g \bigg/ \sum_{g=1}^{G} d_g \qquad \text{Equation 1}$$

Here d_g is the duration of period *g* in the study and n_g is the number of children with congenital rubella infection who had been infected during period *g* in the pregnancy that were followed up. Table 7 summarizes the values for d_g, n_g and r_g for the three studies.

Table 7: Data from the three studies that were used to generate the distribution of the risk of probable CRS given maternal rubella infection during the first 16 weeks of pregnancy

	Time of infection (week of pregnancy) (*g*)	Duration of the period in column 1 (d_g)	Number of children with congenital rubella infection that were followed up (n_g)	Risk of probable CRS during follow-up given infection in the mother (r_g)
Miller et al	<11	10	9	0.9
	11-12	2	4	0.33
	13-14	2	12	0.11
	15-16	2	17	0.24
Grillner et al – assumption 1	1-8	8	2	0.4
	9-12	3	4	0.43
	13-14	2	4	0.29
	15-16	2	7	0.10
Grillner et al – assumption 2	1-8	8	2	0.66
	9-12	3	4	0.43
	13-14	2	4	0.29
	15-16	2	7	0.10
Hahne et al – assumption 1	4-10	7	9	0.9
	11-12	2	2	0.5
	14-18	5	2	0
Hahne et al – assumption 2	1-10	7	9	0.9
	11-13	2	2	0.5
	14-18	5	2	0

For the study of Hahne et al[35], the risk, as calculated using Equation 1, reflects the average during the first 18 weeks of pregnancy, excluding weeks 1-3 and 13, since the study had no data on pregnant women infected during those weeks. Also data for weeks 14-18 were not broken down into data for weeks 14-16 and >17 weeks. In sensitivity analyses

(denoted as assumption 2 for Hahne et al), we recompute the CRS risk assuming that the risks during weeks 1-3 and 13 were the same as those in weeks 4-8 and 11-12 respectively.

As shown in Table 8, for Miller et al the estimated risk of a child being born with probable CRS if the mother had been infected during the first 16 weeks of pregnancy was 65% (95% CI: 49-77%). For Grillner et al it was lower at 37% (assumption 1) or 49% (assumption 2), although the 95% confidence intervals were very wide (6-72% and 9-77% respectively) and overlapped with those of Miller et al. Similarly, for Hahne et al, the estimates for assumption 2 were consistent with those for Miller et al, and the confidence interval for assumption 1 overlapped with those for Miller et al. The actual values in Hahne et al may be an underestimate of the risk during 16 weeks, given that it considered 18 weeks, and the risk after 16 weeks has been found to be small in many studies[38].

The resulting distribution of bootstrap replicates for the risk of CRS following rubella infection in the mother during the first 16 weeks of pregnancy for all three studies is summarized in Figure S3 The distribution of bootstrap values of this CRS risk from Miller et al was generally consistent with that for the gamma distribution (shape and scale parameters of 37 and 56 respectively) assumed in our analyses, with the values from Hahne et al and over 50% of the values from Grillner et al (assumption 2) also falling within the range of the assumed Gamma distribution.

Table 8: Estimated risk of a child being born with probable CRS following rubella infection in the first 16 (Miller et al and Grillner et al) or 18 (Hahne et al) weeks of pregnancy

**Study**	**Estimated risk (%) of probable CRS (95% CI)**
Miller et al	65.5 (48.7,76.6)
Grillner et al, assumption 1	37.5 (6.3,72.3)
Grillner et al, assumption 2	48.7 (9.4,77.2)
Hahne et al, assumption 1	55.6 (41.9,69.2)
Hahne et al, assumption 2	61.1 (45.8,76.5)

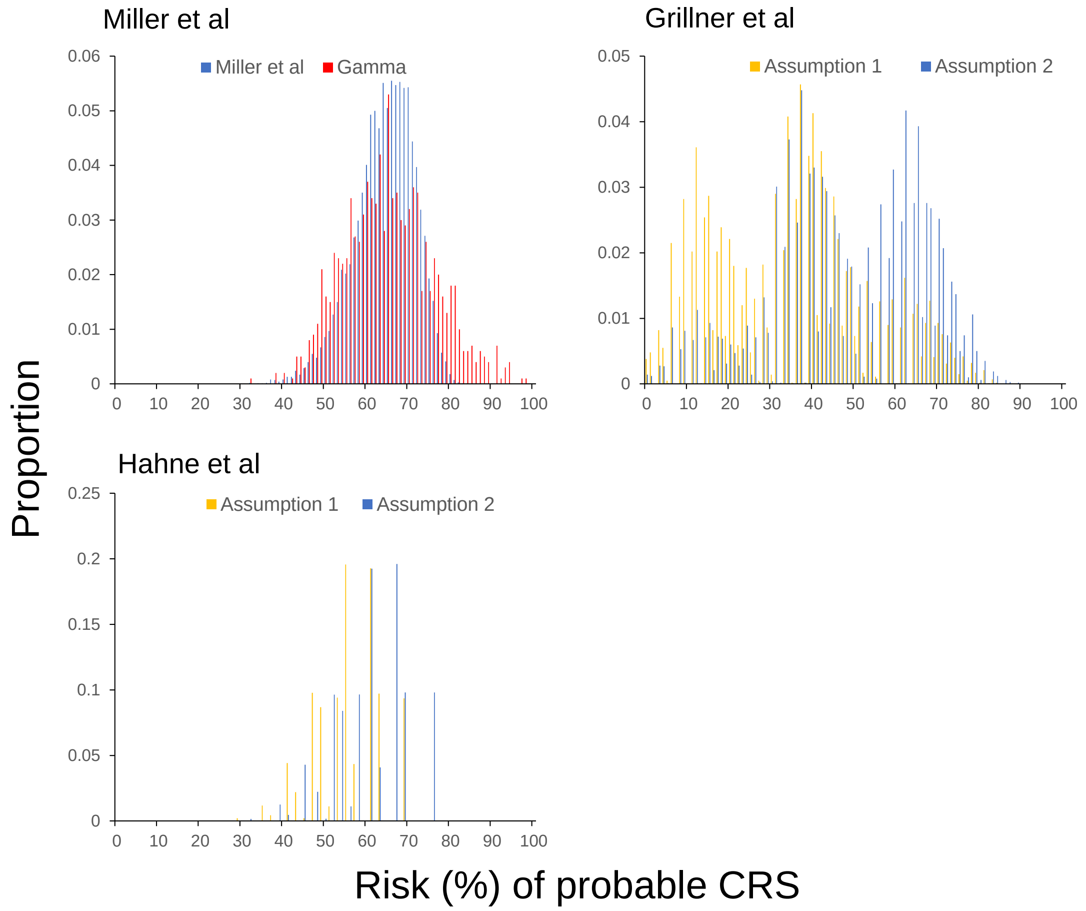

Figure S3: Distribution of the bootstrap replicates of the risk of a child being born with probable CRS following rubella infection in the mother during the first 16 (Miller et al and Grillner et al) or 18 (Hahne et al) weeks of pregnancy. See Table 6, Table 7 and Table 8 for further details about the assumptions for Grillner et al and Hahne et al. The top left figure also shows the outcome of taking 1000 random samples from the Gamma distribution with shape and scale parameters 37 and 56 respectively.

Table 9: Method for compiling 1000 values for the pre-vaccination force of infection for a given country according to the number of seroprevalence datasets available for that country. Further details of the actual datasets used are in Table 10 and Table 11.

Number of available datasets	Method
>1	Drawn from 1000 bootstrap-derived-seroprevalence datasets compiled using equal numbers of bootstrap-derived values from each original dataset, or proportionately to the urban and rural population size, where possible (Table 11).
1	Equal to the 1000 bootstrap-derived-seroprevalence dataset for the single available dataset (Table 11)
0	Obtained by sampling all bootstrap-derived values from the same WHO region (Table 10 and Table 11), with equal numbers of bootstrap-derived values from each country.<br><br>For countries in WPR, as elsewhere[26], the 1000 bootstrap-derived force of infection estimates for a given country without seroprevalence data excluded the force of infection estimates obtained from seroprevalence datasets from China and Australia, as seroprevalence data from these two countries were likely to be atypical of the region.

Table 10: Datasets used to set up 1000 force of infection bootstrap files for the WHO Regions. These bootstrap files were used to generate 1000 contact parameters for use in the transmission model to calculate the median and 95% range of the CRS burden for countries in a given region which did not have any pre-vaccination seroprevalence data (see main text). Note that these datasets had been accepted after performing the selection procedure described in section B. Table 11 provides the datasets for each country.

**Region**	**Datasets**
African (AFR)	Algeria, 2005-7[39], Benin, 1993[40]; Burkina Faso, 2007-8[41]; Cameroon, 2016 (Bafoussam)[42] and <2018 (Yaounde) [43]; Congo, <1991[44]; Cote d'Ivoire, 1975[45] & 1985-6[46]; Democratic Republic of the Congo, 2008-9 (Kikwit, Mikalayi, Tshikapa, Vanga) [27]; Ethiopia, 1981[47] & 1994[48], 2015-17 (Amhara)[69], 2016 (Hawassa)[49] ; Gabon, 1985[50]; Ghana, 1997[51]; Kenya, 1996-9 (Kilifi)[52, 53], 2005 (Eldoret); Madagascar, 1990-1995[54]; Mozambique, 2002[55]; Namibia, 2010 [56]; Nigeria, <1978[57], <2002[58] & 2007-8[59], 2011-12 (Kaduna) [60], 2012 (Ilorin) [61], 2013 (Maiduguri) [62], 2015 (Kaduna) [63]; Senegal, 1996-2001[64]; South Africa, 2003[65], 2014-16 (Soweto) [66]; Tanzania, 2012-13 (Mwanza); Zambia, 1979-80[67],
American, excluding Caribbean (AMR, excl Caribbean)	Argentina, 1967-8 (urban & rural)[68], & 1981 (Mar de Plata)[69]; Brazil, 1967-8[68], 1987[70] & 1996-8[71]; Canada, <1967[72]; Chile 1967-8 (Santiago & rural)[68] and 1983[73]; Mexico, 1987-88[74] & 1989[75]; Panama 1967-8 (Panama City & rural)[68]; Peru, 1967-8 (Lima & rural)[68] & 2003[76]; Uruguay, 1967-7 (urban and rural)[68]; USA <1967 (Atlanta & Houston)[72].
Caribbean	Haiti, 2003[77], Jamaica, 1967-8 (Kingston & rural)[68], Trinidad 1966-7[78], 1967-8 (Port au Spain & rural)[68]
Eastern Mediterranean (EMR)	Bahrain, 1981[79]; Egypt (Cairo), 1973[80]; Iran, 1993-95[81]; Jordan, 1982-3[82]; Kuwait, <1978[83]; Lebanon, 1980-1[84]; Morocco, 1969-70[85]; Pakistan, <1997[86], 1999-2004[87], 2Saudi Arabia, 1989[88] & 1992-93[89]; Sudan, 2015-16 (Khartoum) [90], 2016 (Khartoum) [91]; Tunisia, <1970[92]; Yemen, 1985[93] & 2002-03[94]
European (EUR)	Czech Republic, <1967[72] & 1984 (Prague) [95]; Denmark, <1967[72] &1983[96]; East Germany, 1990[97]; England, <1967[72] & 1986-7[98]; Finland, 1979[99]; France, <1967[72]; Kyrgyzstan, 1968-70[100] & 2001[101]; Poland, 1969[102], 1973[102], 1979 (urban)[103], 1979 (rural)[103], 1982 (urban)[102], 1982 (rural)[102]; Romania, <1989[104]; Spain, 1969-71[105]; Switzerland, 1985[106]; Turkey, 1998[107], 2003-04[108] & 2005[109].
South East Asian (SEAR)	Bangladesh, 2004-5[110]; India, 1968 (urban & rural Delhi)[111], 1972-3 (Chandrigarh & Lucknow)[111], 1976 (Calcutta)[112], <1987 (Delhi)[113], <1990 (Delhi)[114], 1999-2000 (urban and rural Vellore)[115], 2016 (Kerala) [116], 2017 [117]; Indonesia, 2007 (*S Reef, personal communication);* Nepal, 2008[118], Thailand, 1978[119]
Western Pacific (WPR), excluding China & Australia	Fiji, <1973[120]; Japan, <1967 (Sapporo &Ohtsu)[72]; Laos, 2014 [121]; Malaysia, <1972[122]; Singapore, 1975-79[123], Taiwan, 1984[124] & 1984-6[125]; Central Vietnam, 2009-2010[126]

Table 11: Summary of the bootstrap datasets used to establish the pre-vaccination force of infection for each country using the transmission model, using the WHO regional grouping to assign datasets for countries without serological datasets from before the introduction of RCV. See Table 10 for the datasets used to make up the bootstrap datasets.

Country	Bootstrap dataset used:
**Africa**	
Algeria	Algeria, 2005-7[39]
Angola	AFR
Benin	Benin, 1993[40]
Botswana	AFR
Burkina Faso	Burkina Faso, 2007-8[41]
Burundi	AFR
Cameroon	Cameroon, 2016 (Bafoussam) [42] & (Yaounde), <2018[43]
Cape Verde	AFR
Central African Republic	AFR
Chad	AFR
Comoros	AFR
Congo	AFR
Côte d'Ivoire	Cote d'Ivoire, 1975[45] & 1985-6[46]
Democratic Republic of the Congo	Democratic Republic of the Congo, 2008-9 (Kikwit, Mikalayi, Tshikapa, Vanga) [27]
Equatorial Guinea	AFR
Eritrea	AFR
Ethiopia	Ethiopia, 1981[47], 1994[48], 2015-17 (Amhara) [127], 2016 (Hawassa) [49]
Gabon	Gabon, 1985[50]
Gambia	AFR
Ghana	Ghana, 1997[51]
Guinea	AFR
Guinea-Bissau	AFR

Kenya	Kenya (Kilifi), 1996-9[52, 53], 2005 (Eldoret) [128]
Lesotho	AFR
Liberia	AFR
Madagascar	Madagascar, 1990-1995 [54]
Malawi	AFR
Mali	AFR
Mauritania	AFR
Mauritius	AFR
Mozambique	Mozambique, 2002[55]
Namibia	Namibia, 2010[56]
Niger	AFR
Nigeria	Nigeria, <1978[57], <2002[58] & 2007-8[59], 2011-12 (Kaduna) [60], 2012 (Ilorin) [61], 2013 (Maiduguri) [62], 2015 (Kaduna) [63]
Réunion	AFR
Rwanda	AFR
Sao Tome and Principe	AFR
Senegal	Senegal, 1996-2001 [64]
Seychelles	AFR
Sierra Leone	AFR
South Africa	South Africa, 2003 [65], 2014-16 (Soweto) [66]
South Sudan	AFR
Swaziland	AFR
Togo	AFR
Uganda	AFR
United Republic of Tanzania	Tanzania (Mwanza), 2012-13[129]
Western Sahara	AFR
Zambia	Zambia, 1979-80 [67]
Zimbabwe	AFR

Americas	
Antigua and Barbuda	Caribbean
Argentina	Argentina, 1967-8 (urban & rural)[68], & 1981 (Mar de Plata)[69]
Aruba	Caribbean
Bahamas	Caribbean
Barbados	Caribbean
Belize	Caribbean
Bolivia	AMR, excluding the Caribbean
Brazil	Brazil, 1967-8[68], 1987[70] & 1996-8[71]
Canada	Canada, <1967[72]
Chile	Chile 1967-8 (Santiago & rural)[68] & 1983 (Santiago) [73]
Colombia	AMR, excluding the Caribbean
Costa Rica	AMR, excluding the Caribbean
Cuba	Caribbean
Dominican Republic	Caribbean
Ecuador	AMR, excluding the Caribbean
El Salvador	AMR, excluding the Caribbean
French Guiana	Caribbean
Grenada	Caribbean
Guadeloupe	Caribbean
Guatemala	AMR, excluding the Caribbean
Guyana	Caribbean
Haiti	Haiti, 2003[77]
Honduras	AMR, excluding the Caribbean
Jamaica	Jamaica, 1967-8 (Kingston & rural)[68]
Martinique	Caribbean
Mexico	Mexico, 1987-88[74] & 1989[75]

Nicaragua	AMR, excluding the Caribbean
Panama	Panama 1967-8 (Panama City & rural)[68]
Paraguay	AMR, excluding the Caribbean
Peru	Peru, 1967-8 (Lima & rural)[68] & 2003[76]
Puerto Rico	USA (Atlanta and Houston), <1967[72]
Saint Lucia	Caribbean
Saint Vincent and the Grenadines	Caribbean
Suriname	Caribbean
Trinidad and Tobago	Trinidad 1966-7[78], 1967-8 (Port au Spain & rural)[68]
USA	USA <1967 (Atlanta & Houston)[72]
US Virgin Islands	USA <1967 (Atlanta & Houston)[72]
Uruguay	Uruguay, 1967-7 (urban and rural)[68]
Venezuela	AMR, excluding the Caribbean
**Eastern Mediterranean**	
Afghanistan	EMR
Bahrain	Bahrain, 1981[79]
Djibouti	EMR
Egypt	Egypt (Cairo), 1973[80]
Iran	Iran, 1993-95[81]
Iraq	EMR
Jordan	Jordan, 1982-3[82]
Kuwait	Kuwait, <1978[83]
Lebanon	Lebanon, 1980-81[84]
Libya	EMR
Morocco	Morocco, 1969-1970[85]
Sudan	Sudan (Khartoum), 2015-16[90] & 2016[91]
Oman	EMR

Pakistan	Pakistan, <1997[86], 1999-2004[87]
Qatar	EMR
Saudi Arabia	Saudi Arabia, 1989[88] & 1992-3[89]
Somalia	EMR
State of Palestine	EMR
Syrian Arab Republic	EMR
Tunisia	Tunisia, <1970[92]
United Arab Emirates	EMR
Yemen	Yemen, 1985[93] & 2002-3[94]
**Europe**	
Albania	EUR
Armenia	EUR
Austria	EUR
Azerbaijan	EUR
Belarus	EUR
Belgium	EUR
Bosnia and Herzegovina	EUR
Bulgaria	EUR
Channel Islands	EUR
Croatia	EUR
Cyprus	EUR
Czech Republic	Czech Republic, <1967[72] & 1984 (Prague) [95]
Denmark	Denmark, <1967[72] &1983[96]
Estonia	EUR
Finland	Finland, 1979[99]
France	France, <1967[72]
Georgia	EUR

Germany	East Germany, 1990[97]
Greece	EUR
Hungary	EUR
Iceland	EUR
Ireland	EUR
Israel	EUR
Italy	EUR
Kazakhstan	EUR
Kyrgyzstan	Kyrgyzstan, 1968-70[100] & 2001[101]
Latvia	EUR
Lithuania	EUR
Luxembourg	EUR
Malta	EUR
Montenegro	EUR
Netherlands	EUR
Norway	EUR
Poland	Poland, 1969[102], 1973[102], 1979 (urban)[103], 1979 (rural)[103], 1982 (urban)[102], 1982 (rural)[102]
Portugal	EUR
Moldova	EUR
Romania	Romania, <1989[104]
Russia	EUR
Serbia	EUR
Slovakia	EUR
Slovenia	EUR
Spain	Spain, 1969-71[105]
Sweden	EUR
Switzerland	Switzerland, 1985[106]

Macedonia	EUR
Tajikistan	EUR
Turkey	Turkey, 1998[107], 2003-04[108] & 2005[109]
Turkmenistan	EUR
Ukraine	EUR
United Kingdom	England, <1967[72] & 1986-87[98]
Uzbekistan	EUR
**South East Asia**	
Bangladesh	Bangladesh, 2004-5[110]
Bhutan	SEAR
India	India, 1968 (urban & rural Delhi)[111], 1972-3 (Chandrigarh & Lucknow)[111], 1976 (Calcutta)[112], <1987 (Delhi)[113], <1990 (Delhi)[114], 1999-2000 (urban and rural Vellore)[115], 2016 (Kerala) [116], 2017[117]
Indonesia	Indonesia, 2007 (*S Reef, personal communication)*
Korea, Democratic People's Republic	SEAR
Maldives	SEAR
Myanmar	SEAR
Nepal	Nepal, 2008[118]
Sri Lanka	SEAR
Thailand	Thailand, 1978[119]
Timor-Leste	SEAR
**Western Pacific**	
Australia	Australia, <1967[72]
Brunei Darussalam	WPR, excluding China & Australia
Cambodia	WPR, excluding China & Australia
China	China, 1979-80[130]
China (Hong Kong)	China, 1979-80[130]

China (Macao)	China, 1979-80[130]
Fiji	Fiji, <1973[120]
French Polynesia	WPR, excluding China & Australia
Guam	WPR, excluding China & Australia
Japan	Japan, <1967 (Sapporo &Ohtsu)[72]
Kiribati	WPR, excluding China & Australia
Laos	Laos, 2014 [121]
Malaysia	Malaysia, <1972[122]
Micronesia (Fed. States)	WPR, excluding China & Australia
Mongolia	WPR, excluding China & Australia
New Caledonia	WPR, excluding China & Australia
New Zealand	Australia, <1967[72]
Papua New Guinea	WPR, excluding China & Australia
Philippines	WPR, excluding China & Australia
Republic of Korea	WPR, excluding China & Australia
Samoa	WPR, excluding China & Australia
Singapore	Singapore, 1975-9[123]
Solomon Islands	WPR, excluding China & Australia
Taiwan	WPR, excluding China & Australia
Tonga	WPR, excluding China & Australia
Vanuatu	WPR, excluding China & Australia
Vietnam	Central Vietnam, 2009-2010[126]

E: Equations for the CRS incidence and annual numbers of CRS cases

For a given model run, *j*, out of the 1000 model runs, the number of CRS cases per 100,000 livebirths for a given country, *c*, for each year *y* during 1996-2019 was calculated using the following equation

$$I^{B}_{CRS,c,j}(A_{15-49},y) = \frac{N_{CRS,c,j}(A_{15-49},y)}{\sum_{a=15}^{49} f(a,y)N_w(a,y)} \times 100{,}000$$

Here, the denominator is the number of births in year *y* for all women aged 15-49 years, calculated using the fertility rate (*f(a,y)*) and population size ($N_w(a,y)$) of women aged *a* in year *y* in the UN population data and the numerator ($N_{CRS,c,j}(A_{15-49},y)$) is the estimated number of CRS cases born to women aged 15- 49 years in year *y* for the jth set of parameter values. The latter number ($N_{CRS,c,j}(A_{15-49},y)$) was calculated by summing the daily number of CRS cases born to women aged 15-49 years, as follows:

$$N_{CRS,c,j}(A_{15-49},y) = \sum_{t=1}^{365}\sum_{a=15}^{49} \frac{r_{CRS,j}s_{w,j}(a,t)f(a,y)N_w(a,y)(1-e^{-112\lambda_o(t)})}{365}$$

Here, $s_{w,j}(a,t)$ is the modelled proportion of women aged *a* on day *t* that are susceptible, $\lambda_{o,j}(t)$ is the daily model-generated force of infection among women on day t, and $r_{CRS,j}$ is the risk of a newborn of a mother infected during the first 16 weeks of pregnancy having CRS for the jth bootstrap set of values. As elsewhere[26, 131, 132], we assume that infection during the first 16 weeks of pregnancy carries an average 65% (95% range: (47,88%)) risk of the newborn having CRS.

Regional and global estimates

The regional median and 95% CI of the CRS incidence per 100,000 live births was calculated using country-specific estimates (see above), weighted by the population size.

The equation for the jth model run of the average CRS incidence per 100,000 live births for the N countries in a given region was as follows:

$$\frac{\sum_{c=1}^{N} I_{CRS,c,j}^{B}(A_{15-49}, y) P_c(y)}{\sum_{c=1}^{N} P_c(y)}$$

where $I_{CRS,c,j}^{B}(A_{15-49}, y)$ is defined above and $P_c(y)$ is the population size of country c in year y. As previously, given China's large population size, the regional incidence for WPR was calculated with and without excluding China, .

We summed the annual numbers for the jth set of bootstrap parameter values for each country in the region to obtain the corresponding regional totals, which were summed to obtain the global burden (i.e. including all countries). These calculations were repeated for each of the 1000 combinations of parameter values.

The 95% CI of the national, regional and global numbers of CRS cases were approximated by the 95% range of the corresponding 1000 values. The country-specific central value was taken as the median from 1000 model runs if the country had either no or >1 seroprevalence dataset. Otherwise, it was taken as that derived from the estimated prevaccination force of infection from the observed data.

F. Estimates of the pre-vaccination force of infection

Table 12: Summary of the additional datasets that were identified since the previous systematic review, best-fitting values for the force of infection and (where appropriate) the sensitivity of the antibody assay for each catalytic model before the introduction of RCV. The values in parentheses reflect the 95% confidence intervals, obtained by bootstrapping. Several of the estimates were published in an interim update of the literature review[28] and are included here for completeness.

Country, year of study	Study population	Sample size (no. of age groups)	Lab test (cut-off)	Cata-lytic model	Force of infection (/1000/year)		Sensitivity (%)	Loglike-lihood deviance (deg of freedom)	Selected model
					<13 yr olds	≥13 yr olds			
**African region**									
Algeria, 2005-7[39]	Women of child-bearing age	834(6)	ELISA, 10IU	A	0 (0,942)	503 (0,867)	69 (66,100)	2 (3)	
				B	93 (74,99)	0 (0,12)	--	3 (4)	
				C	192 (93,933)	192 (93,933)	69 (66,74)	3 (4)	
				D	36 (33,39)	36 (33,39)	--	32 (5)	D
Burkina Faso, 2007-8[41]	Pregnant F	341(4)	ELISA	A	0 (0,915)	828 (0,1000)	96 (94,100)	2(1)	B
				B	242 (135,282)	3 (0,128)	-	2(2)	
				C	235 (139,990)	235 (139,990)	96 (93,99)	2(2)	
				D	126 (108,154)	126 (108,154)	-	10(3)	
Cameroon (Bafoussam), 2016[42]	pregnant women	91(5)	ELISA, IgG Index >=1.00	A	212 (109,978)	4 (0,836)	100 (90,100)	4 (2)	
				B	212 (109,300)	4 (0,96)	--	4 (3)	
				C	999 (94,999)	999 (94,999)	93 (88,100)	4 (3)	
				D	102 (78,151)	102 (78,151)	--	6 (4)	A

Country, year of study	Study population	Sample size (no. of age groups)	Lab test (cut-off)	Cata-lytic model	Force of infection (/1000/year)		Sensitivity (%)	Loglike-lihood deviance (deg of freedom)	Selected model
					<13 yr olds	≥13 yr olds			
Cameroon (Yaounde), <2018[43]	Pregnant women at ANCs	400(6)	ELISA ?, 10IU	A	153 (33,593)	51 (0,1000)	100 (91,100)	2 (3)	
				B	153 (95,215)	51 (0,123)	--	2 (4)	
				C	149 (102,999)	149 (102,999)	95 (91,100)	2 (4)	
				D	106 (93,123)	106 (93,123)	--	4 (5)	B
Democratic Republic of the Congo (Kikwit), 2008-9[27]	Pregnant F	254 (5)	ELISA, ≥10IU	A	145 (103,632)	27 (0,75)	100 (89,100)	5(2)	B
				B	145 (105,189)	27 (0,69)	-	5(3)	
				C	999 (83,999)	999 (83,999)	89 (85,100)	6(3)	
				D	86 (74,103)	86 (74,103)	-	10(4)	
Democratic Republic of the Congo (Mikalayi), 2008-9[27]	Pregnant F	206 (5)	ELISA, ≥10IU	A	0 (0,466)	557 (0,992)	82 (77,100)	0(2)	B
				B	103 (66,138)	23 (0,68)	-	1(3)	
				C	125 (67,969)	125 (67,969)	84 (76,99)	1(3)	
				D	63 (54,77)	63 (54,77)	-	6(4)	

Country, year of study	Study population	Sample size (no. of age groups)	Lab test (cut-off)	Cata-lytic model	Force of infection (/1000/year)		Sensitivity (%)	Loglike-lihood deviance (deg of freedom)	Selected model
					<13 yr olds	≥13 yr olds			
Democratic Republic of the Congo (Tshikapa), 2008-9[27]	Pregnant F	182 (5)	ELISA, ≥10IU	A	128 (75,913)	20 (0,248)	100 (82,100)	58 (0,187)	B
				B	128 (84,169)	20 (0,73)	-	58 (0,180)	
				C	168 (77,999)	168 (77,999)	86 (80,100)	58 (0,200)	
				D	76 (62,94)	76 (62,94)	-	202 (163,232)	
Democratic Republic of the Congo (Vange), 2008-9[27]	Pregnant F	255 (5)	ELISA, ≥10IU	A	132 (90,252)	32 (0,75)	100 (91,100)	75 (0,165)	B
				B	132 (91,178)	32 (0,72)	-	75 (0,164)	
				C	968 (77,999)	968 (77,999)	87 (84,100)	0 (0,200)	
				D	83 (71,97)	83 (71,97)	-	187 (157,211)	
Ethiopia (Amhara), 2015-17[127]	Pregnant women	600(5)	EIA, >10IU	A	113 (0,425)	19 (10,815)	100 (78,100)	0 (2)	
				B	108 (72,136)	17 (0,51)	--	2 (3)	
				C	108 (76,837)	108 (76,837)	88 (78,93)	1 (3)	
				D	60 (54,67)	60 (54,67)	--	9 (4)	B

Country, year of study	Study population	Sample size (no. of age groups)	Lab test (cut-off)	Cata -lytic mod el	Force of infection (/1000/year)		Sensitivity (%)	Loglike-lihood deviance (deg of freedom)	Selected model
					<13 yr olds	≥13 yr olds			
Ethiopia (Hawassa), 2016[49]	Pregnant women at ANCs	422(5)	ELISA	A	0 (0,987)	985 (0,1000)	86 (83,97)	2 (2)	
				B	159 (130,179)	0 (0,26)	--	3 (3)	
				C	606 (142,989)	606 (142,989)	84 (83,91)	3 (3)	
				D	73 (70,89)	73 (70,89)	--	18 (4)	D
Kenya (Eldoret), 2005[128]	Pregnant F	437(4)	EIA, ≥10IU	A	140 (0,219)	147 (24,875)	97 (93,100)	0(1)	B
				B	154 (91,223)	66 (1,152)	-	1(2)	
				C	142 (107,950)	142 (107,950)	97 (93,100)	0(2)	
				D	113 (100,131)	113 (100,131)	-	2(3)	
Namibia, 2010[56]	Pregnant women attending ANCS	2040(6)	EIA, OD>0. 2	A	146 (0,167)	24 (10,705)	100 (90,100)	7 (3)	B
				B	146 (127,167)	24 (10,39)	--	7 (4)	
				C	168 (126,894)	168 (126,894)	91 (89,93)	11 (4)	
				D	81 (76,86)	81 (76,86)	--	60 (5)	

Country, year of study	Study population	Sample size (no. of age groups)	Lab test (cut-off)	Cata-lytic model	Force of infection (/1000/year)		Sensitivity (%)	Loglike-lihood deviance (deg of freedom)	Selected model
					<13 yr olds	≥13 yr olds			
Nigeria (Kaduna), 2011-12[60]	pregnant women attending ANCs	400(6)	ELISA	A	681 (0,985)	0 (0,1000)	97 (95,100)	1 (3)	
				B	268 (201,313)	0 (0,74)	--	1 (4)	
				C	962 (185,999)	962 (185,999)	97 (95,99)	1 (4)	
				D	137 (118,168)	137 (118,168)	--	13 (5)	D
Nigeria (Ilorin), 2012[61]	women of child-bearing age attending general outpatients department	285(4)	ELISA	A	160 (0,544)	39 (0,663)	100 (93,100)	1 (1)	
				B	160 (104,226)	39 (0,104)	--	1 (2)	
				C	158 (96,995)	158 (96,995)	94 (90,100)	2 (2)	
				D	99 (85,121)	99 (85,121)	--	5 (3)	B
Nigeria (Maiduguri), 2013[62]	Pregnant ANC attendees	90(5)	ELISA	A	118 (0,978)	717 (0,999)	84 (77,99)	5 (2)	D
				B	143 (78,184)	0 (0,65)	--	5 (3)	
				C	311 (91,999)	311 (91,999)	83 (77,95)	5 (3)	
				D	73 (57,96)	73 (57,96)	--	11 (4)	

Country, year of study	Study population	Sample size (no. of age groups)	Lab test (cut-off)	Cata-lytic model	Force of infection (/1000/year)		Sensitivity (%)	Loglike-lihood deviance (deg of freedom)	Selected model
					<13 yr olds	≥13 yr olds			
Nigeria (Kaduna), 2015[63]	Pregnant women attending ANC	900(8)	ELISA	A	132 (14,986)	42 (2,1000)	72 (62,100)	7 (5)	
				B	72 (55,85)	9 (0,24)	--	7 (6)	
				C	135 (71,998)	135 (71,998)	66 (61,77)	7 (6)	
				D	40 (36,44)	40 (36,44)	--	23 (7)	B
Nigeria (Keffi), <2016 [133]	Pregnant women at ANC	220(5)	ELISA, IgG index 1.0	A	359 (0,930)	0 (0,629)	12 (10,100)	2 (2)	
				B	10 (1,14)	0 (0,8)	--	2 (3)	
				C	891 (4,995)	891 (4,995)	12 (8,100)	2 (3)	
				D	5 (3,7)	5 (3,7)	--	4 (4)	D – drop
South Africa (Soweto), 2014-16[66]	Pregnant women during labour or within 24 hours after delivery	552(3)	ELISA, ≥11IU	A	249 (141,382)	71 (0,202)	100 (100,100)	4 (0)	B
				B	249 (141,364)	71 (0,195)	--	4 (1)	
				C	212 (143,963)	212 (143,963)	99 (98,100)	5 (1)	
				D	160 (139,199)	160 (139,199)	--	6 (2)	

Country, year of study	Study population	Sample size (no. of age groups)	Lab test (cut-off)	Cata-lytic model	Force of infection (/1000/year)		Sensitivity (%)	Loglike-lihood deviance (deg of freedom)	Selected model
					<13 yr olds	≥13 yr olds			
Tanzania (Mwanza), 2012-13[129]	Pregnant F	342 (3)	EIA, ≥10IU	A	0 (0,226)	544 (20,760)	96 (93,100)	0(0)	B
				B	142 (74,216)	79 (6,190)	-	1(1)	
				C	135 (105,265)	135 (105,265)	98 (93,100)	0(1)	
				D	115 (99,137)	115 (99,137)	-	1(2)	
**American region**									
Chile (Santiago), 1983[73]	Pregnant women	812(5)	ELISA	A	0 (0,283)	866 (8,799)	95 (94,100)	2 (2)	B
				B	182 (131,236)	45 (0,104)	--	2 (3)	
				C	164 (121,971)	164 (121,971)	96 (94,100)	2 (3)	
				D	115 (105,129)	115 (105,129)	--	9 (4)	
**Eastern Mediterranean region**									
Egypt (Cairo), 1973[80]	Healthy population (well-baby clinic attendees, schoolchildren, students & women at gynaecology & obstetrics	402(7)	HAI	A	1000 (631,1000)	121 (71,171)	88 (85,91)	27 (4)	B
				B	175 (150,210)	49 (14,86)	--	64 (5)	
				C	1000 (637,1000)	1000 (637,1000)	88 (85,91)	27 (5)	
				D	148 (129,175)	148 (129,175)	--	70 (6)	

Country, year of study	Study population	Sample size (no. of age groups)	Lab test (cut-off)	Cata-lytic model	Force of infection (/1000/year)		Sensitivity (%)	Loglike-lihood deviance (deg of freedom)	Selected model
					<13 yr olds	≥13 yr olds			
Sudan (Khartoum), 2015-16[90]	General population	447(6)	ELISA	A	123 (100,147)	1000 (714,1000)	95 (92,98)	10 (3)	B
				B	129 (108,150)	51 (24,99)	--	17 (4)	
				C	136 (113,159)	136 (113,159)	96 (92,99)	16 (4)	
				D	107 (92,126)	107 (92,126)	--	27 (5)	
Sudan (Khartoum), 2016[91]	Healthy pregnant women	92(3)	EIA, >10IU	A	194 (0,992)	8 (0,914)	100 (88,100)	0 (0)	B
				B	195 (53,274)	8 (0,136)	--	0 (1)	
				C	270 (82,945)	270 (82,945)	92 (88,100)	0 (1)	
				D	86 (67,125)	86 (67,125)	--	3 (2)	
**Europe**									
Czech (Prague), 1984[95]	Pregnant women	850(4)	HI test	A	130 (42,181)	57 (18,241)	100 (94,100)	1 (1)	A
				B	130 (80,177)	57 (16,110)	--	1 (2)	
				C	111 (89,750)	111 (89,750)	97 (91,100)	2 (2)	
				D	92 (85,101)	92 (85,101)	--	4 (3)	

Country, year of study	Study population	Sample size (no. of age groups)	Lab test (cut-off)	Cata-lytic model	Force of infection (/1000/year)		Sensitivity (%)	Loglike-lihood deviance (deg of freedom)	Selected model
					<13 yr olds	≥13 yr olds			
Kyrgyzstan, 1968-70[100]	Donor sites and maternity hospitals	1629(3)	HI test	A	148 (28,176)	70 (17,240)	95 (91,100)	0 (0)	A
				B	140 (114,169)	30 (11,51)	--	0 (1)	
				C	125 (101,180)	125 (101,180)	93 (90,96)	0 (1)	
				D	82 (77,88)	82 (77,88)	--	22 (2)	
Poland, 1969[102]	girls and women	1087(6)	?	A	182 (153,217)	320 (136,818)	96 (95,99)	4 (3)	B
				B	174 (151,199)	85 (43,139)	--	9 (4)	
				C	186 (158,221)	186 (158,221)	97 (95,99)	5 (4)	
				D	151 (138,165)	151 (138,165)	--	18 (5)	
Poland, 1973[102]	girls and women	1066(5)	?	A	161 (137,189)	620 (289,1000)	95 (93,97)	3 (2)	B
				B	157 (138,177)	91 (49,145)	--	12 (3)	
				C	170 (147,197)	170 (147,197)	97 (94,99)	9 (3)	
				D	141 (130,154)	141 (130,154)	--	17 (4)	
Poland (urban), 1979[103]	Randomly selected sites	866(10)	HI test, 1:10	A	149 (128,175)	135 (92,231)	99 (97,100)	13 (7)	A
				B	148 (129,171)	101 (65,153)	--	14 (8)	
				C	148 (130,175)	148 (130,175)	99 (97,100)	13 (8)	
				D	137 (122,153)	137 (122,153)	--	16 (9)	

Country, year of study	Study population	Sample size (no. of age groups)	Lab test (cut-off)	Cata-lytic model	Force of infection (/1000/year)		Sensitivity (%)	Loglike-lihood deviance (deg of freedom)	Selected model
					<13 yr olds	≥13 yr olds			
Poland (rural), 1979[103]	Randomly selected sites	763(10)	HI test, 1:10	A	126 (104,145)	314 (103,1000)	97 (95,100)	14 (7)	B
				B	128 (110,148)	117 (76,186)	--	17 (8)	
				C	132 (117,152)	132 (117,152)	99 (97,100)	16 (8)	
				D	126 (113,141)	126 (113,141)	--	17 (9)	
Poland (urban), 1982[102]	Healthy population	666(8)	HI test	A	172 (150,211)	95 (58,1000)	100 (96,100)	6 (5)	B
				B	172 (150,197)	95 (50,169)	--	6 (6)	
				C	179 (151,224)	179 (151,224)	98 (95,100)	7 (6)	
				D	158 (142,178)	158 (142,178)	--	9 (7)	
Poland (rural), 1982[102]	Healthy population	545(8)	HI test	A	157 (135,187)	106 (64,1000)	100 (95,100)	15 (5)	B
				B	157 (135,183)	106 (60,192)	--	15 (6)	
				C	156 (134,199)	156 (134,199)	99 (95,100)	16 (6)	
				D	148 (132,168)	148 (132,168)	--	16 (7)	

Country, year of study	Study population	Sample size (no. of age groups)	Lab test (cut-off)	Cata-lytic model	Force of infection (/1000/year)		Sensitivity (%)	Loglike-lihood deviance (deg of freedom)	Selected model
					<13 yr olds	≥13 yr olds			
Spain, 1969-71[105]	Healthy population at large	1076(9)	HI test	A	378 (300,487)	284 (0,1000)	89 (86,92)	15 (6)	C
				B	204 (188,223)	0 (0,4)	--	41 (7)	
				C	378 (303,489)	378 (303,489)	89 (86,92)	15 (7)	
				D	158 (143,179)	158 (143,179)	--	163 (8)	
Switzerland, 1985[106]	Sera submitted for diagnostic testing unrelated to rubella	736(28)	ELISA, 10IU	A	51 (37,67)	224 (153,318)	92 (89,95)	50 (25)	B
				B	64 (50,80)	64 (46,88)	--	75 (26)	
				C	78 (66,92)	78 (66,92)	95 (92,99)	68 (26)	
				D	64 (58,72)	64 (58,72)	--	75 (27)	
**South East Asia**									
India (Kerala), 2016[116],	Pregnant women	70(3)	ELISA, 15IU	A	0 (0,993)	838 (0,972)	95 (90,100)	1 (0)	D
				B	229 (0,340)	0 (0,448)	--	1 (1)	
				C	343 (109,996)	343 (109,996)	94 (90,100)	1 (1)	
				D	120 (90,196)	120 (90,196)	--	2 (2)	

Country, year of study	Study population	Sample size (no. of age groups)	Lab test (cut-off)	Cata-lytic model	Force of infection (/1000/year)		Sensitivity (%)	Loglike-lihood deviance (deg of freedom)	Selected model
					<13 yr olds	≥13 yr olds			
India, 2017[117]	Pregnant women attending ANC clinic or womens hospital	1800(5)	ELISA, >10iu	A	344 (0,994)	0 (0,966)	86 (83,100)	1 (2)	D
				B	151 (135,159)	0 (0,14)	--	1 (3)	
				C	854 (179,994)	854 (179,994)	85 (83,87)	1 (3)	
				D	73 (69,78)	73 (69,78)	--	45 (4)	
Indonesia, 2007 (*S Reef, personal communication, March 2015*)	General population	11320 (10)	?	A	135 (119,148)	62 (32,97)	93 (91,97)	3(7)	B
				B	127 (120,135)	22 (18,26)	-	12(8)	
				C	115 (106,126)	115 (106,126)	91 (90,92)	12(8)	
				D	61 (60,63)	61 (60,63)	-	462(9)	
**Western Pacific**									
Cambodia, 2012[134]	Nationwide cross-section of women aged 15-39 years	2154(5)	ElISA (OD >0.2	A	64 (0,100)	191 (71,421)	80 (77,87)	1(2)	B
				B	76 (65,89)	33 (21,44)	--	8(3)	
				C	91 (74,116)	91 (74,116)	84 (79,89)	3(3)	
				D	54 (51,58)	54 (51,58)	--	23(4)	

Country, year of study	Study population	Sample size (no. of age groups)	Lab test (cut-off)	Cata-lytic model	Force of infection (/1000/year)		Sensitivity (%)	Loglike-lihood deviance (deg of freedom)	Selected model
					<13 yr olds	≥13 yr olds			
Laos, 2014 [121]*	General population aged >21 years(i.e. excluding the age range vaccinated in the SIA in 2011)	1013 (45)	ELISA, >10IU	A	90 (38,141)	18 (9,127)	100 (82,100)	60 (42)	B
				B	90 (69,111)	18 (8,29)	-	60 (43)	
				C	80 (58,129)	80 (58,129)	85 (79,91)	61 (43)	
				D	44 (40,47)	44 (40,47)	-	78 (44)	

* The methods for calculating the force of infection for Laas are identical to those presented in [121], except that, for consistency with the estimates for the other countries, the force of infection is assumed to differ between those aged ≤13 years and those aged >13 years.

G. Estimates of the CRS incidence

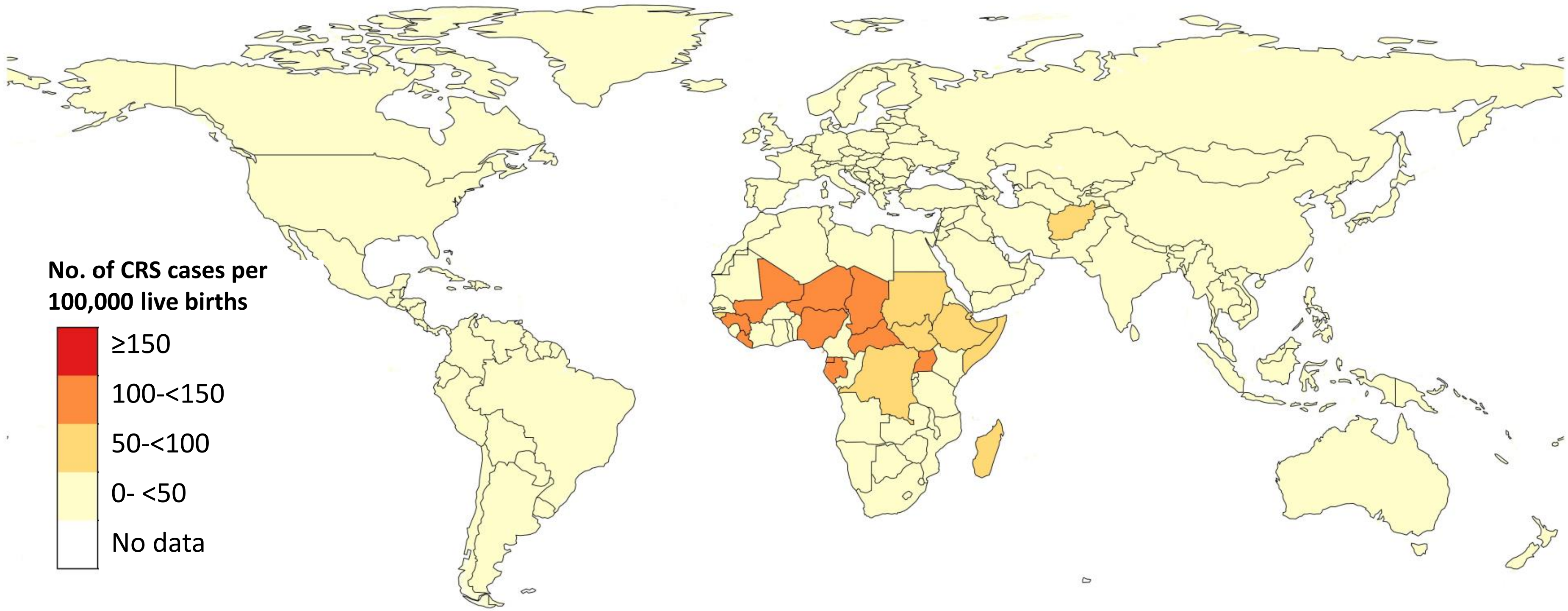

Figure S4: Average estimates of the number of CRS cases per 100,000 live births in 2019 for all countries.

Table 13: The median CRS incidence per 100,000 live births and number of CRS cases born in each WHO region and worldwide in 1996, 2000, 2010 and 2019 and the percentage of the regional live births occurring in countries which had introduced RCV by these years. The numbers in parentheses reflect 95% confidence limits.

	Year	% of the regional live births in countries with RCV	CRS incidence per 100,000 live births	Total number of CRS cases
**African region**	1996	0.15	121 (64,211)	29468 (14763,52921)
	2000	0.14	121 (63,212)	32073 (15935,57609)
	2010	0.16	119 (62,211)	38873 (19574,70294)
	2019	41	64 (24,123)	25454 (9193,48881)
**Eastern Mediterranean region**	1996	12	62 (33,110)	8440 (3976,15981)
	2000	30	54 (24,109)	8011 (2908,17387)
	2010	45	28 (5,68)	5514 (1116,13043)
	2019	49	27 (4,67)	5660 (873,14073)
**European region**	1996	52	71 (21,176)	8534 (2920,20403)
	2000	68	40 (15,101)	5682 (2092,13184)
	2010	100	4 (1,18)	319 (52,1545)
	2019	100	1 (0,12)	100 (0,957)
**Region of the Americas**	1996	61	58 (26,112)	11626 (5241,22202)
	2000	90	12 (6,26)	2657 (1170,5562)
	2010	100	<1 (0,3)	1 (0,357)
	2019	100	<1 (0,1)	<1 (0,90)
**South East Asian region**	1996	3	126 (30,247)	50035 (10866,99711)
	2000	3	123 (28,250)	49404 (10379,101215)
	2010	3	121 (26,245)	45444 (9316,92325)
	2019	100	<1 (<1,8)	51 (<1,1662)
**Western Pacific Region (excluding China)**	1996	41	123 (59,234)	11741 (5501,22734)
	2000	40	113 (56,214)	10925 (5235,19887)
	2010	69	101 (50,199)	10006 (4700,19258)
	2019	100	<1 (<1,44)	<1 (<1,3756)
**Western Pacific Region (including China)**	1996	12	33 (16,61)	12179 (5887,22940)
	2000	12	31 (16,58)	11486 (5569,20502)
	2010	91	27 (13,52)	10023 (4704,19261)
	2019	100	<1 (0,12)	<1 (0,3768)
**Global**	1996	16	-	120687 (70405,191204)
	2000	23	-	109353 (61616,179222)
	2010	42	-	99665 (53554,165778)
	2019	77	-	32460 (12794,59818)

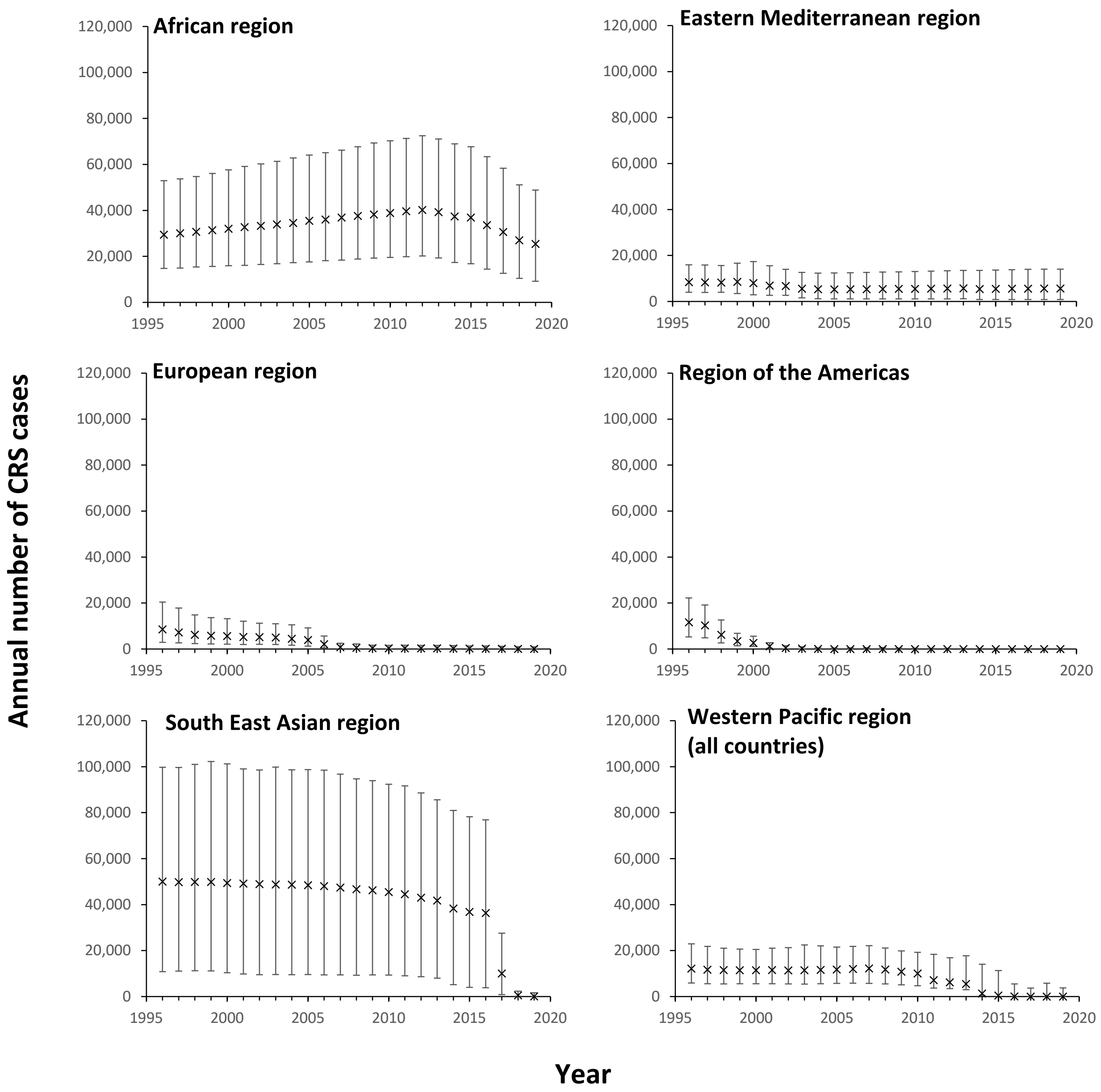

Figure S5: Estimates of the number of CRS cases born annually in each WHO region during 1996-2019. The error bars show the 95% range (CI).

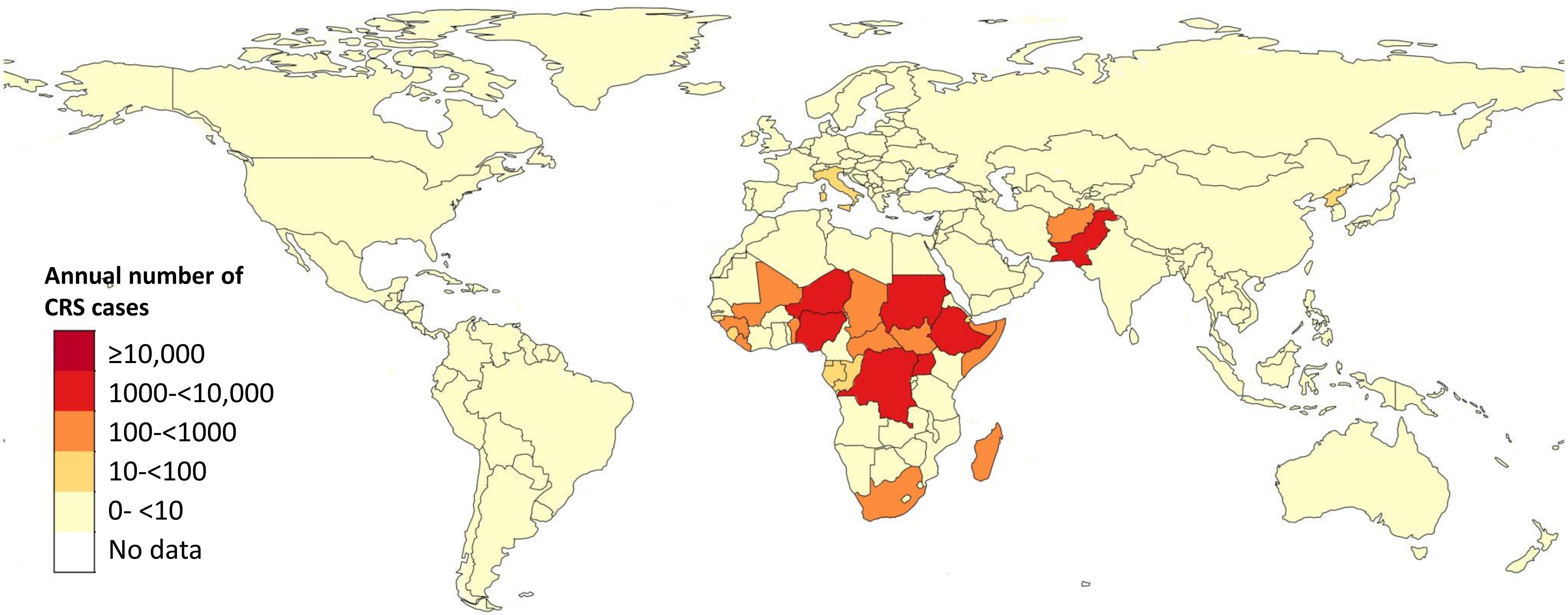

Figure S6: Estimates of the average number of CRS cases born annually in each country in 2019

Table 14: Comparison between the estimated number of CRS cases in 2010, 2019 and during 2011-2019 with vaccination as implemented, and the numbers that might have been expected if vaccination had not been introduced after 2010. The final column shows the estimated number of CRS cases averted during 2011-2019 by the introduction of RCV after 2010.

	Year	Number of CRS cases		Total number of CRS cases during 2011-2019		Number averted during 2011-2019 by RCV introduced after 2010
		**RCV as implemented**	**RCV not introduced after 2010**	**RCV as implemented**	**RCV not introduced after 2010**	
**African region**	**2010**	38873 (19574,70294)	38873 (19574,70294)	-	-	-
	**2019**	25454 (9193,48881)	44146 (22018,79076)	349510 (162083,648091)	414344 (209074,746863)	65370 (37446,111474)
**Eastern Mediterranean region**	**2010**	5514 (1116,13043)	5514 (1116,13043)	-	-	-
	**2019**	5660 (873,14073)	5976 (1195,14348)	55976 (9773,135945)	57895 (11580,137674)	1504 (417,3323)
**South East Asian region**	**2010**	45444 (9316,92325)	45444 (9316,92325)	-	-	-
	**2019**	51 (0,1662)	40036 (8699,81023)	297575 (50447,627451)	422457 (89519,866180)	124896 (37346,234769)
**Western Pacific Region**	**2010**	10023 (4704,19261)	10023 (4704,19261)	-	-	-
	**2019**	<1 (0,3768)	5829 (3412,11251)	31362 (16089,114954)	67706 (37839,152574)	35741 (21030,59327)
**Global**	**2010**	99665 (53554,165778)	99665 (53554,165778)	-	-	-
	**2019**	32460 (12794,59818)	96271 (53937,157435)	743197 (392673,1254921)	969653 (531016,1626758)	228969 (130929,367995)

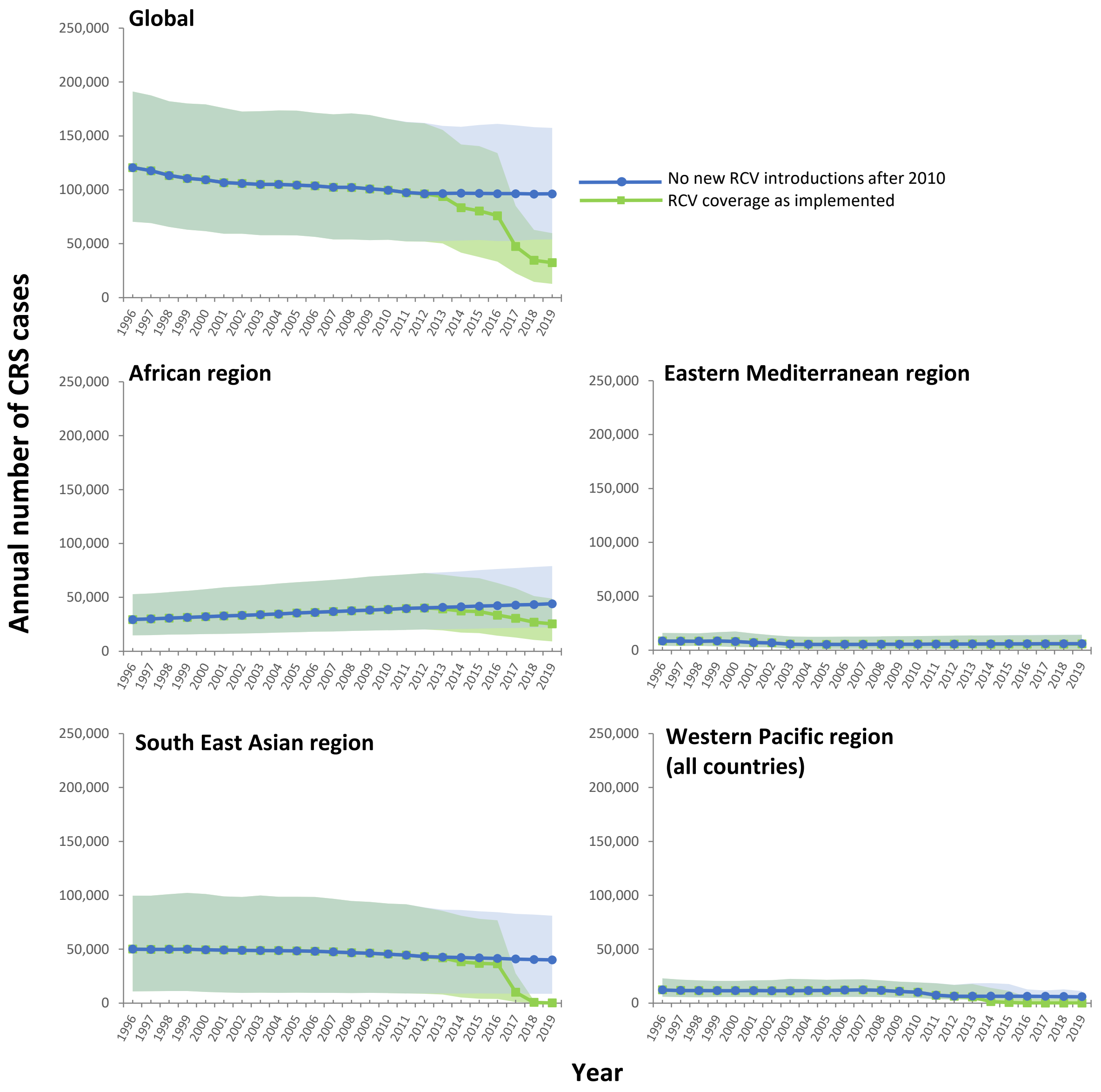

Figure S7: Estimates of the annual number of CRS cases during 1996-2019 globally, in the African, Eastern Mediterranean, South East Asian regions, as calculated using the RCV coverage as implemented and the number that might have been seen if there had been no new introductions of RCV after 2010. The shaded areas show the 95% confidence intervals.

References

1. Turnbull FM, Burgess MA, McIntyre PB, Lambert SB, Gilbert GL, Gidding HF, et al. The Australian Measles Control Campaign, 1998. Bull World Health Organ. 2001;79(9):882-8.
2. Heywood AE, Gidding HF, Riddell MA, McIntyre PB, MacIntyre CR, Kelly HA. Elimination of endemic measles transmission in Australia. Bull World Health Organ. 2009;87(1):64-71.
3. Gidding HF. The impact of Australia's measles control programme over the past decade. Epidemiol Infect. 2005;133(1):99-105.
4. Kearns MJ, Plitt SS, Lee BE, Robinson JL. Rubella immunity among pregnant women in a Canadian provincial screening program. Can J Infect Dis Med Microbiol. 2009;20(3):73-7.
5. Furesz J. Elimination of measles in the Americas. CMAJ. 1996;155(10):1423-6.
6. Haargaard B, Wohlfahrt J, Fledelius HC, Rosenberg T, Melbye M. A nationwide Danish study of 1027 cases of congenital/infantile cataracts: etiological and clinical classifications. Ophthalmology. 2004;111(12):2292-8.
7. Glismann S. Rubella in Denmark. Euro Surveill. 2004;9(4):12-3.
8. Ueda K. Development of the rubella vaccine and vaccination strategy in Japan. Vaccine. 2009;27(24):3232-3.
9. Narimah A. Rubella immunization in Malaysia--20 years on, and the challenges ahead. Med J Malaysia. 2005;60(3):267-8.
10. Ministry of Health. Immunisation handbook. Wellington; 2006.
11. Reid S. Evolution of the New Zealand childhood immunisation schedule from 1980: a personal view. N Z Med J. 2006;119(1236):U2035.
12. Palihawadana P, Wickremasinghe AR, Perera J. Seroprevalence of rubella antibodies among pregnant females in Sri Lanka. Southeast Asian J Trop Med Public Health. 2003;34(2):398-404.
13. Bottiger M, Forsgren M. Twenty years' experience of rubella vaccination in Sweden: 10 years of selective vaccination (of 12-year-old girls and of women postpartum) and 13 years of a general two-dose vaccination. Vaccine. 1997;15(14):1538-44.
14. Preblud SR, Serdula MK, Frank JA, Jr., Brandling-Bennett AD, Hinman AR. Rubella vaccination in the United States: a ten-year review. Epidemiol Rev. 1980;2:171-94.
15. Progress toward elimination of rubella and congenital rubella syndrome--the Americas, 2003-2008. MMWR Morb Mortal Wkly Rep. 2008;57(43):1176-9.
16. CAREC. Rubella vaccination. In: CAREC, editor. 1995.
17. CAREC. Rubella mass campaigns. 2001.
18. Anis E, Grotto I, Moerman L, Kaliner E, Warshavsky B, Slater PE, et al. Rubella in Israel after the MMR vaccine: elimination or containment? J Public Health Policy. 2013;34(2):288-301.
19. Levine H, Ankol OE, Rozhavski V, Davidovitch N, Aboudy Y, Zarka S, et al. Rubella seroprevalence in the first birth cohort reaching fertility age after 20 years of two dose universal vaccination policy in Israel. Vaccine. 2012;30(50):7260-4.
20. Tharmaphornpilas P, Yoocharean P, Rasdjarmrearnsook AO, Theamboonlers A, Poovorawan Y. Seroprevalence of antibodies to measles, mumps, and rubella among Thai population: evaluation of measles/MMR immunization programme. J Health Popul Nutr. 2009;27(1):80-6.
21. World Health Organization. Measles and rubella elimination country profile. Hungary [Available from: https://www.euro.who.int/__data/assets/pdf_file/0013/401116/HUN.pdf?ua=1.
22. Taiwan Centers for Disease Control. Rubella [Available from: https://www.cdc.gov.tw/En/Category/ListContent/bg0g_VU_Ysrgkes_KRUDgQ?uaid=5OqfXlJc1CDMqKnytW3Qww.
23. Nardone A, Tischer A, Andrews N, Backhouse J, Theeten H, Gatcheva N, et al. Comparison of rubella seroepidemiology in 17 countries: progress towards international disease control targets. Bull World Health Organ. 2008;86(2):118-25.

24. Robertson SE, Cutts FT, Samuel R, Diaz-Ortega JL. Control of rubella and congenital rubella syndrome (CRS) in developing countries, Part 2: Vaccination against rubella. Bull World Health Organ. 1997;75(1):69-80.
25. Robertson SE, Featherstone DA, Gacic-Dobo M, Hersh BS. Rubella and congenital rubella syndrome: global update. Rev Panam Salud Publica. 2003;14(5):306-15.
26. Vynnycky E, Adams EJ, Cutts FT, Reef SE, Navar AM, Simons E, et al. Using Seroprevalence and Immunisation Coverage Data to Estimate the Global Burden of Congenital Rubella Syndrome, 1996-2010: A Systematic Review. PLoS One. 2016;11(3):e0149160.
27. Alleman MM, Wannemuehler KA, Hao L, Perelygina L, Icenogle JP, Vynnycky E, et al. Estimating the burden of rubella virus infection and congenital rubella syndrome through a rubella immunity assessment among pregnant women in the Democratic Republic of the Congo: Potential impact on vaccination policy. Vaccine. 2016;34(51):6502-11.
28. Vynnycky E, Papadopoulos T, Angelis K. The impact of Measles-Rubella vaccination on the morbidity and mortality from Congenital Rubella Syndrome in 92 countries. Hum Vaccin Immunother. 2019;15(2):309-16.
29. Schenzle D. An age-structured model of pre- and post-vaccination measles transmission. IMA J Math Appl Med Biol. 1984;1(2):169-91.
30. UN Statistics Division UNPD. World Population Prospects. 2019.
31. Abbey H. An examination of the Reed-Frost theory of epidemics. Hum Biol. 1952;24(3):201-33.
32. van den Boogaard J, de Gier B, de Oliveira Bressane Lima P, Desai S, de Melker HE, Hahne SJM, et al. Immunogenicity, duration of protection, effectiveness and safety of rubella containing vaccines: A systematic literature review and meta-analysis. Vaccine. 2021;39(6):889-900.
33. Miller E, Cradock-Watson JE, Pollock TM. Consequences of confirmed maternal rubella at successive stages of pregnancy. Lancet. 1982;2(8302):781-4.
34. Grillner L, Forsgren M, Barr B, Bottiger M, Danielsson L, De Verdier C. Outcome of rubella during pregnancy with special reference to the 17th-24th weeks of gestation. Scand J Infect Dis. 1983;15(4):321-5.
35. Hahne S, Macey J, van Binnendijk R, Kohl R, Dolman S, van der Veen Y, et al. Rubella outbreak in the Netherlands, 2004-2005: high burden of congenital infection and spread to Canada. Pediatr Infect Dis J. 2009;28(9):795-800.
36. Simons EA, Reef SE, Cooper LZ, Zimmerman L, Thompson KM. Systematic Review of the Manifestations of Congenital Rubella Syndrome in Infants and Characterization of Disability-Adjusted Life Years (DALYs). Risk Anal. 2014.
37. Cutts FT, Vynnycky E. Modelling the incidence of congenital rubella syndrome in developing countries. Int J Epidemiol. 1999;28(6):1176-84.
38. Thompson KM, Simons EA, Badizadegan K, Reef SE, Cooper LZ. Characterization of the Risks of Adverse Outcomes Following Rubella Infection in Pregnancy. Risk Anal. 2016;36(7):17.
39. Ouyahia A, A.; S, Laouamri S, Touabti A, Lacheheb A. Seroprevalence of rubella among women of childbearing age in Algeria. International Journal of Public Health and Epidemiology Vol 2 (1) pp 056-059, January, 2013. 2013;2(1):4.
40. Rodier MH, Berthonneau J, Bourgoin A, Giraudeau G, Agius G, Burucoa C, et al. Seroprevalences of Toxoplasma, malaria, rubella, cytomegalovirus, HIV and treponemal infections among pregnant women in Cotonou, Republic of Benin. Acta tropica. 1995;59(4):271-7.
41. Tahita MC, Hubschen JM, Tarnagda Z, Ernest D, Charpentier E, Kremer JR, et al. Rubella seroprevalence among pregnant women in Burkina Faso. BMC Infect Dis. 2013;13:164.
42. Noubom M, Tseyep DGK, Sama LF, Wam EC, Sadjeu S, Kenfack B, et al. Seroprevalence of Rubella IgM and IgG Antibodies and Associated Risk Factors among Pregnant Women Attending Antenatal Clinic at Bafoussam Regional Hospital, West Region of Cameroon. J Trop Dis. 2018;6(4):7.

43. Eno AA, Makemgue LS, Tonye DS, III, Boye CSB, Seck A, Bercion R, et al. Seroprevalence of rubella-specific IgM and IgG antibodies among pregnant women in a catholic hospital in Yaounde, Cameroon. International Journal of Biological and Chemical Sciences. 2018;12(3):1123-30.
44. Yala F, Biendo M, Odongo I, Kounkou R. [Virological and bacteriological study of materno-fetal infections in Brazzaville]. Bulletin de la Societe de pathologie exotique (1990). 1991;84(5 Pt 5):627-34.
45. Vrinat M, Dutertre J, Helies H, Ropero P. [A serological survey of rubella among pregnant women in Abidjan (author's transl)]. Medecine tropicale : revue du Corps de sante colonial. 1978;38(1):53-7.
46. Ouattara SA, Brettes JP, Kodjo R, Penali K, Gershy-Damet G, Sangare A, et al. [Seroepidemiology of rubella in the Ivory Coast. Geographic distribution]. Bulletin de la Societe de pathologie exotique et de ses filiales. 1987;80(4):655-64.
47. Sandow D, Okubagzhi GS, Arnold U, Denkmann N. Seroepidemiological study in rubella in pregnant women in Gondar Region, northern Ethiopia. Ethiopian medical journal. 1982;20(4):173-8.
48. Cutts FT, Abebe A, Messele T, Dejene A, Enquselassie F, Nigatu W, et al. Sero-epidemiology of rubella in the urban population of Addis Ababa, Ethiopia. Epidemiol Infect. 2000;124(3):467-79.
49. Tamirat B, Hussen S, Shimelis T. Rubella virus infection and associated factors among pregnant women attending the antenatal care clinics of public hospitals in Hawassa City, Southern Ethiopia: A cross-sectional study. BMJ open. 2017;7(10).
50. Mefane C. Rubella antibodies in 1737 girls and women in Gabon. Afrique Medicale. 1985;24(226):29-32.
51. Lawn JE, Reef S, Baffoe-Bonnie B, Adadevoh S, Caul EO, Griffin GE. Unseen blindness, unheard deafness, and unrecorded death and disability: congenital rubella in Kumasi, Ghana. American journal of public health. 2000;90(10):1555-61.
52. Cumberland P, Shulman CE, Maple PA, Bulmer JN, Dorman EK, Kawuondo K, et al. Maternal HIV infection and placental malaria reduce transplacental antibody transfer and tetanus antibody levels in newborns in Kenya. J Infect Dis. 2007;196(4):550-7.
53. Scott S, Cumberland P, Shulman CE, Cousens S, Cohen BJ, Brown DW, et al. Neonatal measles immunity in rural Kenya: the influence of HIV and placental malaria infections on placental transfer of antibodies and levels of antibody in maternal and cord serum samples. J Infect Dis. 2005;191(11):1854-60.
54. Dromigny JA, Pecarrere JL, Ollivier G, Leroy F, Zeller HG. [Seroprevalence of rubella in pregnant women at Antananarivo. Study of 853 sera at the Pasteur Institute in Madagascar]. Archives de l'Institut Pasteur de Madagascar. 1996;63(1-2):53-5.
55. Barreto J, Sacramento I, Robertson SE, Langa J, de Gourville E, Wolfson L, et al. Antenatal rubella serosurvey in Maputo, Mozambique. Trop Med Int Health. 2006;11(4):559-64.
56. Jonas A, Cardemil CV, Beukes A, Anderson R, Rota PA, Bankamp B, et al. Rubella immunity among pregnant women aged 15-44 years, Namibia, 2010. International journal of infectious diseases : IJID : official publication of the International Society for Infectious Diseases. 2016;49:196-201.
57. Odelola HA. Rubella haemagglutination inhibiting antibodies in females of child-bearing age in western Nigeria. Journal of hygiene, epidemiology, microbiology, and immunology. 1978;22(2):190-4.
58. Bukbuk DN, el Nafaty AU, Obed JY. Prevalence of rubella-specific IgG antibody in non-immunized pregnant women in Maiduguri, north eastern Nigeria. Central European journal of public health. 2002;10(1-2):21-3.
59. Amina MD, Oladapo S, Habib S, Adebola O, Bimbo K, Daniel A. Prevalence of rubella IgG antibodies among pregnant women in Zaria, Nigeria. International Health. 2010;2(2):156-9.
60. Mangga HK, Aminu M, Inabo HI. Seroprevalence of rubella specific IgG antibody among pregnant women attending ante-natal clinics in Kaduna metropolis, Nigeria. Nigerian Journal of Basic and Applied Sciences. 2014;22(3/4):63-6.

61. Suleiman ST, Bakare R, Akanbi AA, Fowotade A, Billiaminu SA, Durowade KA. Rubella Antibody Avidity Among Rubella Seropositive Women Attending a Tertiary Care Facility in Nigeria. Int J Womens Health Reprod Sci. 2015;3(3):136-41.
62. Oyinloye SO, Amama CA, Daniel R, Ajayi BB, Lawan MA. Seroprevalence survey of rubella antibodies among pregnant women in Maiduguri, Borno state, Nigeria. African Journal of Clinical and Experimental Microbiology. 2014;15(3):151-7.
63. Gubio AB, Olonitola S, Jattau E, Mukhtar MA, editors. Sero-prevalence of rubella virus among pregnant women in Kaduna State Nigeria 2015. ISDS 2016; 2016: Online Journal of Public health Informatics; 2017.
64. Dromigny JA, Nabeth P, Perrier Gros Claude JD. Evaluation of the seroprevalence of rubella in the region of Dakar (Senegal). Trop Med Int Health. 2003;8(8):740-3.
65. Corcoran C, Hardie DR. Seroprevalence of rubella antibodies among antenatal patients in the Western Cape. South African medical journal = Suid-Afrikaanse tydskrif vir geneeskunde. 2005;95(9):688-90.
66. Gieles NC, Mutsaerts E, Kwatra G, Bont L, Cutland CL, Jones S, et al. Rubella seroprevalence in pregnant women living with and without HIV in Soweto, South Africa. International journal of infectious diseases : IJID : official publication of the International Society for Infectious Diseases. 2020;91:255-60.
67. Watts T. Rubella antibodies in a sample of Lusaka mothers. Medical journal of Zambia. 1983;17(4):109-10.
68. Dowdle WR, Ferrera W, De Salles Gomes LF, King D, Kourany M, Madalengoitia J, et al. WHO collaborative study on the sero-epidemiology of rubella in Caribbean and Middle and South American populations in 1968. Bull World Health Organ. 1970;42(3):419-22.
69. Pereira F, Uez O. Rubella antibodies in female applicants for premarital health certificates in Mar del Plata, Argentina. Bull Pan Am Health Organ. 1986;20(2):179-85.
70. Souza VA, Moraes JC, Sumita LM, Camargo MC, Fink MC, Hidalgo NT, et al. Prevalence of rubella antibodies in a non-immunized urban population, Sao Paulo, Brazil. The Division of Immunization, CVE. Revista do Instituto de Medicina Tropical de Sao Paulo. 1994;36(4):373-6.
71. Reiche EM, Morimoto HK, Farias GN, Hisatsugu KR, Geller L, Gomes AC, et al. [Prevalence of American trypanosomiasis, syphilis, toxoplasmosis, rubella, hepatitis B, hepatitis C, human immunodeficiency virus infection, assayed through serological tests among pregnant patients, from 1996 to 1998, at the Regional University Hospital Norte do Parana]. Rev Soc Bras Med Trop. 2000;33(6):519-27.
72. Rawls WE, Melnick JL, Bradstreet CM, Bailey M, Ferris AA, Lehmann NI, et al. WHO collaborative study on the sero-epidemiology of rubella. Bull World Health Organ. 1967;37(1):79-88.
73. Vial P, Torres-Pereyra J, Stagno S, Gonzalez F, Donoso E, Alford CA, et al. Serologic screening for cytomegalovirus, rubella virus, herpes simplex virus, hepatitis B virus, and Toxoplasma gondii in two urban populations of pregnant women in Chile. Bull Pan Am Health Organ. 1986;20(1):53-61.
74. Gutierrez Trujillo G, Munoz O, Tapia Conyer R, Bustamante Calvillo ME, Alvarez y Munoz MT, Guiscafre Gallardo JP, et al. [The seroepidemiology of rubella in Mexican women. A national probability survey]. Salud publica de Mexico. 1990;32(6):623-31.
75. Yamamoto L, Mejia E, Lopez RM, Gallardo E, Gomez B. Susceptibility to rubella infection in females at high risk. Immune protection associated to population density. Tropical and geographical medicine. 1995;47(6):235-8.
76. Suarez-Ognio L, Adrianzen A, Ortiz A, Martinez C, Whittembury A, Cabezudo E, et al. A rubella serosurvey in postpartum women in the three regions of Peru. Rev Panam Salud Publica. 2007;22(2):110-7.
77. Desinor OY, Anselme RJ, Laender F, Saint-Louis C, Bien-Aime JE. Seroprevalence of antibodies against rubella virus in pregnant women in Haiti. Rev Panam Salud Publica. 2004;15(3):147-50.

78. Pitts OM, Ravenel JM, Finklea JF. Rubella immunity in Trinidad. American journal of epidemiology. 1969;89(3):271-6.
79. Dutta SR, Atrash HK, Mathew L, Mathew PP, Mahmood RA. Seroepidemiology of rubella in Bahrain. Int J Epidemiol. 1985;14(4):618-23.
80. Khalifa AS, Abdel-Wahab KS, Imam IZ. Seroimmunity to rubella among an Egyptian population. J Infect Dis. 1973;127(5):567-70.
81. Modarres S, Modarres S, Oskoii NN. The immunity of children and adult females to rubella virus infection in Tehran. Iranian journal of medical sciences. 1996;21:69-73.
82. El-Khateeb MS, Tarawneh MS, Hijazi S, Kahwaji L. Seroimmunity to rubella virus in Jordanians. Public Health. 1983;97(4):204-7.
83. Hathout H, Al-Nakib W, Lilley H, Abo-Ahmed HS, Nosseir AF. Seroepidemiology of rubella in Kuwait: an alternative vaccination policy. Int J Epidemiol. 1978;7(1):49-53.
84. Bedrossian NK, Matossian R. Is there a rubella problem in Lebanon? Lebanese Medical Journal. 1985;35(1):31-8.
85. Nejmi S. [Immunologic survey of rubella in Moroccan women in the Rabat region (study of antibodies inhibiting hemagglutination in 548 serums)]. Maroc medical. 1972;52(559):420-5.
86. Iqbal A, Bokhari S. Occurrence of rubella antibody IgG in the general population. Mother and Child. 1997;35(1):17-22.
87. Ahmed R, Hashmi K, Ullah SE, Khanum T, Rafia A. Study of Prevalence of Immune Status in Adult Females For Rubella Virus Infection. Pakistan Journal of Biological Sciences. 2006;9(5):816.
88. Hossain A. Seroepidemiology of rubella in Saudi Arabia. Journal of tropical pediatrics. 1989;35(4):169-70.
89. Saeed A, Abu-Shagra S, Al-Rasheed R. Congenital Rubella Syndrome - revisited (letter). Saudi Med J. 1993;14(25-26).
90. Adam O, Musa A, Kamer A, Sausy A, Tisserand E, Hubschen JM. Seroprevalence of measles, mumps, and rubella and genetic characterization of mumps virus in Khartoum, Sudan. International Journal of Infectious Diseases. 2020;91:87-93.
91. Abass E, Abduljalil W, Alnour S, Mohammed T, Hamid S, Mostafa S, et al. Seroprevalence of Rubella Antibodies in Infertile and Pregnant Sudanese Women. Journal of Clinical and Diagnostic Research. 2018;12(12):4.
92. Nabli B. [Seroepidemiology of rubella in Tunisia]. Bull World Health Organ. 1970;42(6):891-6.
93. Strauss J, Dobahi SS, Danes L, Kopecky K, Svandova E. Serological survey of rubella in Yemen in 1985. Journal of hygiene, epidemiology, microbiology, and immunology. 1989;33(2):163-7.
94. Sallam TA, Al-Jaufy AY, Al-Shaibany KS, Ghauth AB, Best JM. Prevalence of antibodies to measles and rubella in Sana'a, Yemen. Vaccine. 2006;24(37-39):6304-8.
95. Strauss J, Mrazova M, Macatova I, Zastera M, Svandova M, Smelhausova M, et al. [Risk of teratogenic infections during pregnancy in mothers in Prague]. Cas Lek Cesk. 1986;125(51):1581-6.
96. Glikmann G, Petersen I, Mordhorst CH. Prevalence of IgG-antibodies to mumps and measles virus in non-vaccinated children. Dan Med Bull. 1988;35(2):185-7.
97. Edmunds WJ, Gay NJ, Kretzschmar M, Pebody RG, Wachmann H. The pre-vaccination epidemiology of measles, mumps and rubella in Europe: implications for modelling studies. Epidemiol Infect. 2000;125(3):635-50.
98. Morgan-Capner P, Wright J, Miller CL, Miller E. Surveillance of antibody to measles, mumps, and rubella by age. BMJ. 1988;297(6651):770-2.
99. Ukkonen P. Rubella immunity and morbidity: impact of different vaccination programs in Finland 1979-1992. Scand J Infect Dis. 1996;28(1):31-5.
100. Andzhaparidze OG, Desiatskova RG, Chervonskii GI, Aminova MG, Termetchikova NM. [The seroepidemiology of rubella in the USSR]. Vopr Virusol. 1972;17(4):412-8.
101. Malakmadze N, Zimmerman LA, Uzicanin A, Shteinke L, Caceres VM, Kasymbekova K, et al. Development of a rubella vaccination strategy: contribution of a rubella susceptibility study of

women of childbearing age in Kyrgyzstan, 2001. Clinical infectious diseases : an official publication of the Infectious Diseases Society of America. 2004;38(12):1780-3.
102. Imbs D, Rudnicka H, Diuwe A. [Seroepidemiologic studies of rubella among the population in Poland]. Przegl Epidemiol. 1985;39(2):193-8.
103. Imbs D, Rudnicka H, Prus A. [Rubella virus antibodies among girls and women in Poland]. Przegl Epidemiol. 1980;34(3):241-7.
104. Dumitrescu R, Mateescu M, Gaicu N, Comanescu D. Evaluation of the anti-rubella immunity levels on a lot of 5,000 sera from women at procreative age, tested by HAI, in Romania. Archives roumaines de pathologie experimentales et de microbiologie. 1989;48(3):253-63.
105. Najera E, Najera R, Gallardo FP. The seroepidemiology of rubella: the independent analysis of current prevalence and past incidence in serological surveys in Spain. Bull World Health Organ. 1973;49(1):25-30.
106. Matter L, Germann D, Bally F, Schopfer K. Age-stratified seroprevalence of measles, mumps and rubella (MMR) virus infections in Switzerland after the introduction of MMR mass vaccination. European journal of epidemiology. 1997;13(1):61-6.
107. Aksakal FN, Maral I, Cirak MY, Aygun R. Rubella seroprevalence among women of childbearing age residing in a rural region: is there a need for rubella vaccination in Turkey? Jpn J Infect Dis. 2007;60(4):157-60.
108. Pehlivan E, Karaoglu L, Ozen M, Gunes G, Tekerekoglu MS, Genc MF, et al. Rubella seroprevalence in an unvaccinated pregnant population in Malatya, Turkey. Public Health. 2007;121(6):462-8.
109. Sasmaz T, Kurt AO, Ozturk C, Bugdayci R, Oner S. Rubella seroprevalence in women in the reproductive period, Mersin, Turkey. Vaccine. 2007;25(5):912-7.
110. Nessa A, Islam MN, Tabassum S, Munshi SU, Ahmed M, Karim R. Seroprevalence of rubella among urban and rural Bangladeshi women emphasises the need for rubella vaccination of pre-pubertal girls. Indian journal of medical microbiology. 2008;26(1):94-5.
111. Seth P, Manjunath N, Balaya S. Rubella infection: the Indian scene. Rev Infect Dis. 1985;7 Suppl 1:S64-7.
112. Chakravarty MS, Gupta B, Das BC, Mukherjee MK, Mitra AC, Sarkar JK. Seroepidemiological study of rubella in Calcutta. The Indian journal of medical research. 1976;64(1):87-92.
113. Khare S, Banerjee K, Padubidri V, Rai A, Kumari S, Kumari S. Lowered immunity status of rubella virus infection in pregnant women. The Journal of communicable diseases. 1987;19(4):391-5.
114. Khare S, Gupta HL, Banerjee K, Kumari S, Kumari S, Gupta HL. Seroimmunity to rubella virus infection in young adult females in Delhi. The Journal of communicable diseases. 1990;22(4):279-80.
115. Brown DWJ, Cutts FT, Joseph A. An evaluation of complementary epidemiological methods in a defined population in Southern India for estimating the burden of Congenital Rubella Syndrome. 2004.
116. Thayyil J, Anitha M, Biju G, Bhaskar R, Kuniyil V. Sero prevalence of rubella immunity in pregnant women in Kerala, India. International Journal of Medicine and Public Health. 2016;6(4):189-92.
117. Muliyil DE, Pratibha S, Jois SK, Suhas O, Vanita S, Vishakha V, et al. Sero-prevalence of rubella among pregnant women in India, 2017. Vaccine. 2018;36(52):7909-12.
118. Upreti SR, Thapa K, Pradhan YV, Shakya G, Sapkota YD, Anand A, et al. Developing rubella vaccination policy in Nepal--results from rubella surveillance and seroprevalence and congenital rubella syndrome studies. J Infect Dis. 2011;204 Suppl 1:S433-8.
119. Desudchit P, Chatiyanonda K, Bhamornsathit S. Rubella antibody among Thai women of childbearing age. Southeast Asian J Trop Med Public Health. 1978;9(3):312-6.
120. Macnamara FN, Mitchell R, Miles JA. A study of immunity to rubella in villages in the Fiji islands using the haemagglutination inhibition test. J Hyg (Lond). 1973;71(4):825-31.
121. Hachiya M, Miyano S, Mori Y, Vynnycky E, Keungsaneth P, Vongphrachanh P, et al. Evaluation of nationwide supplementary immunization in Lao People's Democratic Republic:

Population-based seroprevalence survey of anti-measles and anti-rubella IgG in children and adults, mathematical modelling and a stability testing of the vaccine. PLoS One. 2018;13(3):e0194931.
122. Lam SK. The seroepidemiology of rubella in Kuala Lumpur, West Malaysia. Bull World Health Organ. 1972;47(1):127-9.
123. Doraisingham S, Goh KT. The rubella immunity of women of child-bearing age in Singapore. Annals of the Academy of Medicine, Singapore. 1981;10(2):238-41.
124. Black FL. Measles active and passive immunity in a worldwide perspective. Prog Med Virol. 1989;36:1-33.
125. Yuan CF, Ng HT. Seroepidemiologic study of rubella in Taiwan's female population. American journal of public health. 1988;78(10):1366-7.
126. Miyakawa M, Yoshino H, Yoshida LM, Vynnycky E, Motomura H, Tho le H, et al. Seroprevalence of rubella in the cord blood of pregnant women and congenital rubella incidence in Nha Trang, Vietnam. Vaccine. 2014;32(10):1192-8.
127. Wondimeneh Y, Tiruneh M, Ferede G, Abera B, Workineh M, Birhanie M, et al. Rubella virus infections and immune status among pregnant women before the introduction of rubella vaccine in Amhara Regional State, Ethiopia. International journal of infectious diseases : IJID : official publication of the International Society for Infectious Diseases. 2018;76:14-22.
128. Kombich JM, PC; Borus, PK Seroprevalence of Natural Rubella Antibodies among Antenatal Attendees at Moi Teaching and Referral Hospital, Eldoret, Kenya. Journal of Immunological Techniques in Infectious Diseases. 2012;1(1).
129. Mwambe B, Mirambo MM, Mshana SE, Massinde AN, Kidenya BR, Michael D, et al. Sero-positivity rate of rubella and associated factors among pregnant women attending antenatal care in Mwanza, Tanzania. BMC Pregnancy Childbirth. 2014;14:95.
130. Wannian S. Rubella in the People's Republic of China. Reviews of Infectious Diseases. 1985;7(Supp 1):S72.
131. Vynnycky E, Gay NJ, Cutts FT. The predicted impact of private sector MMR vaccination on the burden of Congenital Rubella Syndrome. Vaccine. 2003;21(21-22):2708-19.
132. Vynnycky E, Yoshida LM, Huyen DT, Trung ND, Toda K, Cuong NV, et al. Modeling the impact of rubella vaccination in Vietnam. Hum Vaccin Immunother. 2016;12(1):150-8.
133. Pennap GR, Egwa MA. Prevalence of Rubella Virus Infection among Pregnant Women Accessing Antenatal Clinic at Federal Medical Centre, Keffi, Nigeria. Int J Curr Microbiol App Sci.2016, 5(6): 171-178.
134. Mao B, Chheng K, Wannemuehler K, Vynnycky E, Buth S, Soeung SC, et al. Immunity to polio, measles and rubella in women of child-bearing age and estimated congenital rubella syndrome incidence, Cambodia, 2012. Epidemiol Infect. 2014:1-10.
